# Nusinersen treatment in adult patients with spinal muscular atrophy: a safety analysis of laboratory parameters

**DOI:** 10.1007/s00415-021-10569-8

**Published:** 2021-04-25

**Authors:** Benjamin Stolte, Michael Nonnemacher, Kathrin Kizina, Saskia Bolz, Andreas Totzeck, Andreas Thimm, Bernd Wagner, Cornelius Deuschl, Christoph Kleinschnitz, Tim Hagenacker

**Affiliations:** 1 Department of Neurology, University Hospital Essen, Hufelandstr. 55, 45147 Essen, Germany; 2 Institute for Medical Informatics, Biometrics and Epidemiology, University Hospital Essen, Essen, Germany; 3 Department of Clinical Chemistry, University Hospital Essen, Essen, Germany; 4 Institute for Diagnostic and Interventional Radiology and Neuroradiology, University Hospital Essen, Essen, Germany; 5 Center for Translational and Behavioral Neuroscience, University Hospital Essen, Essen, Germany

**Keywords:** ASO, Platelets, Kidney, Liver, Coagulation, Toxicity, Side effects

## Abstract

**Background:**

Nusinersen is an intrathecally administered antisense oligonucleotide (ASO) that improves motor function in patients with spinal muscular atrophy (SMA). In addition to efficacy, the safety of a therapy is the decisive factor for the success of the treatment. For some ASOs, various organ toxicities have been described, such as thrombocytopenia, renal and liver impairment, or coagulation abnormalities. However, systematic data on laboratory parameters under treatment with nusinersen are mainly available from studies in infants and children. Therefore, our aim was to assess the safety of nusinersen therapy in adult SMA patients.

**Methods:**

Laboratory data from 404 nusinersen injections performed in 50 adult patients with SMA type 2 and type 3 were retrospectively analyzed.

**Results:**

The total observation period was 76.9 patient-years, and patients received up to 12 injections. Our data provides no new safety concerns. In cerebrospinal fluid (CSF), the mean white blood cell count and lactate remained stable over time. Total CSF protein increased by 2.9 mg/dL. No change in mean platelet count was observed under therapy. Only one patient showed sporadic mild thrombocytopenia. Coagulation parameters and inflammatory markers were stable. The mean creatinine level decreased by 0.09 mg/dL. Analysis of mean liver enzyme levels revealed no relevant changes during treatment.

**Conclusion:**

Our data demonstrate a favorable safety profile of nusinersen therapy in adult SMA patients under longer-term “real-world” conditions. In particular, we found no evidence of clinically relevant platelet declines, coagulopathies, or renal or hepatic organ toxicities, which are common concerns with the use of ASOs.

**Supplementary Information:**

The online version contains supplementary material available at 10.1007/s00415-021-10569-8.

## Introduction

The condition, 5q-associated spinal muscular atrophy (5q-SMA) is a rare genetic neuromuscular disorder that leads to progressive muscle atrophy and weakness because of degeneration of anterior horn cells [28]. The “classic” clinical classification of the disease is based on the achievement of motor milestones during childhood, with a broad range of phenotypes from severely affected infants never achieving the ability to sit (SMA type 1), to individuals able to sit but never achieving the ability to walk (SMA type 2), to ambulatory patients (SMA type 3) [31].

The disorder 5q-SMA is caused by a homozygous deletion or mutation in the survival of the motor neuron 1 gene (*SMN1*) localized on chromosome 5q, which results in insufficient levels of SMN proteins[24, 25]. Nusinersen is an antisense oligonucleotide (ASO) that is capable of increasing SMN protein production.

In clinical trials, intrathecal treatment with nusinersen led to meaningful benefits on motor functions in infants and children[14, 29]. Based on the results of these studies, nusinersen was approved by the US Food and Drug Administration (FDA) and the European Medicines Agency (EMA). Moreover, both the feasibility and efficacy of nusinersen therapy have also been well established in adult SMA patients [18, 26, 37, 39, 42, 43].

In addition to feasibility and efficacy, the safety of a therapy is the decisive factor for the success of the treatment. In the past, various organ toxicities have been described with the use of some ASOs, such as an increase in liver transaminases, renal impairment, coagulation abnormalities, or thrombocytopenia [3, 7, 15, 20]. Since ASOs are not able to cross the blood–brain barrier (BBB), nusinersen is administered intrathecally [16]. In terms of potential side effects, this factor could make a difference compared to other ASOs.

To date, systematic data on laboratory parameters under treatment with nusinersen are still scarce and are mainly available from pivotal studies in infants and children [11, 38]. Here, we present our data on laboratory findings in adult SMA patients under treatment with nusinersen. These data are valuable to better assess the safety of longer-term nusinersen therapy in this patient cohort.

## Materials and methods

### Subjects and data collection

Data from 53 patients with 5q-SMA type 1, 2 or 3 treated with nusinersen (defined as at least one successful injection) in the Department of Neurology at the Essen University Hospital, Germany, were retrospectively analyzed.

Demographic data, clinical findings and patient history were obtained before therapy started. In addition, established motor assessments were performed and included the Revised Upper Limb Module (RULM) and the Hammersmith Functional Motor Scale Expanded (HFMSE).

Intrathecal therapy with nusinersen was carried out according to the recommended dosing schedule with four loading doses (L) on treatment days 0 (L1 = baseline), 14 (L2), 28 (L3), and 63 (L4) followed by maintenance doses (M) every four months (M1…, M8). Each dose comprised 12 mg of nusinersen. Depending on the presence of scoliosis or spondylodesis, intrathecal injections were performed via conventional lumbar punctures on an outpatient basis or under image guidance (fluoroscopy-assisted or computed tomography (CT)-guided) during a short hospital stay. Prior to the injections, 5 mL of cerebrospinal fluid (CSF) was removed, and blood samples were collected for laboratory analyses according to our standard of care.

### CSF analyses

Samples with a relevant increased red blood cell (RBC) count (defined as at least 1000 RBCs per microliter) were excluded from statistical CSF analysis because of possible distortion of the other parameters. The following parameters were statistically evaluated: CSF white blood cell (CSF-WBC) count [number/µL], glucose [mg/dL], lactate [mmol/L] and total protein [mg/dL]. The determination of CSF cell count was performed with the automated hematology analyzer XN 1000 (Sysmex Europe, Norderstedt, Germany) in body fluid mode using fluorescence flow cytometry. A distinction between mononuclear cells and granulocytes was performed in samples with pleocytosis. Glucose (hexokinase-glucose-6-phosphate dehydrogenase method), lactate (lactic dehydrogenase method) and total protein (pyrogallol red-molybdate method) were measured using a Dimension XPand clinical chemistry analyzer (Siemens Healthcare Diagnostics, Eschborn, Germany).

### Blood analyses

Blood samples from outpatients were usually acquired before the first loading dose (L1) and each maintenance dose (M1 to M8). In contrast, in hospitalized patients, blood samples were generally obtained at each stay (L1 to L4 and M1 to M8).

The following parameters were collected from all patients and considered in the statistical evaluation: white blood cells (WBCs) and platelets from the blood count [number/nL] as well as creatinine [mg/dL], urea nitrogen [mg/dL], alanine aminotransferase (ALT) [U/L], aspartate aminotransferase (AST) [U/L], gamma-glutamyltransferase (GGT) [U/L] and C-reactive protein (CRP) [mg/dL] from serum. Blood counts were obtained on an automated hematology analyzer XN 1000 (Sysmex Europe, Norderstedt, Germany) with impedance measurement and fluorescence flow cytometry. Clinical chemistry determinations were performed using Advia Chemistry and Atellica CH 930 analyzers (Siemens Healthcare Diagnostics, Eschborn, Germany). The creatinine assay is based on the Jaffe reaction using picric acid in an alkaline medium [19]. The activities of ALT and AST were measured with assays using pyridoxal-5′-phosphate and alpha-ketoglutarate as reagents. The GGT assay is based on a procedure according to Shaw [35]. The urea nitrogen assay is based on the Roch-Ramel enzymatic reaction using urease and glutamate dehydrogenase [34]. The CRP concentration was determined by an immunoturbidimetric assay.

In addition, before each injection, the activated partial thromboplastin time (aPTT) [s] and the international normalized ratio (INR) as a function of prothrombin time were measured in hospitalized patients as markers of the intrinsic and extrinsic pathways of coagulation, respectively. However, the coagulation values of outpatients were only measured and statistically evaluated in a few cases, as they were mostly collected in external laboratories with different reference values. The aPTT and prothrombin time were measured on CS-5100 analyzers (Sysmex Europe, Norderstedt, Germany) and Atellica COAG 360 (Siemens Healthcare Diagnostics, Eschborn, Germany) analyzers. The aPTT assay uses a soybean and rabbit brain phospholipid solution with a plasma activator with increased sensitivity to lupus anticoagulant-like substances. The prothrombin time assay uses thromboplastin and calcium as clotting activators. The INR is calculated using the lot and device-specific international sensitivity index value.

### Reference values

The following reference values were used: CSF-WBC count 0–5/µL, glucose 49–75 mg/dL, lactate 1.2–2.1 mmol/L, and total protein 15–45 mg/dL. WBC count 3.6–9.2/nL, platelet count 140–380/nL, INR 0.85–1.15, aPTT 24.4–32.4 s, creatinine 0.6–1.3 mg/dL, urea nitrogen 6.0–19.8 mg/dL, AST and ALT < 50 U/L each, GGT < 55 U/L, and CRP < 0.5 mg/dL. It should be noted that some gender-specific reference values (platelet count as well as creatinine and liver values) were unified for the purpose of evaluation.

### Calculation of the mean change from baseline under therapy with nusinersen

To identify trends in laboratory values in the total cohort and SMA subgroups on therapy, the mean change from baseline was calculated for each parameter. To increase the sensitivity for detecting longer-term trends, the last available measured value was compared with the respective baseline value in each patient. In addition, the mean change from baseline was calculated for each injection.

### Counting of deviations from the normal range under therapy with nusinersen

To detect potential toxicities in individual patients, the number of patients with normal baseline values who showed deviations from the normal range under therapy (i.e., shifts to abnormal laboratory values) was counted. The duration of the deviations was divided into three categories: “Sporadic” deviations were defined as only single events with immediate normalization afterwards. “Transient” deviations were defined as two or more deviations in a row with subsequent normalization or two deviations in a row at the end of the observation period. “Persistent” deviations were defined as three or more deviations in a row without normalization until the end of the observation period. If patients met more than one criterion (e.g., a single and two consecutive elevations), they were assigned to the "worst" category. It should be noted that for aPTT and INR, only elevations were considered pathological (increased bleeding risk), while in CSF for lactate, only elevations, and for glucose, only decreases (possible signs of infection) were counted.

### Statistical analyses

Statistical analyses were performed with SAS 9.4 software (SAS Institute Inc., Cary, North Carolina, United States) for Windows (Microsoft Corp., Redmond, Washington, United States). Continuous variables were described by the mean, median, standard deviation, quartiles and range. Categorical data were described by absolute and relative frequencies. The Mann–Whitney *U* test or Kruskal–Wallis test was used for comparison of subgroups, and the sign test was used for the analysis of changes between injections. To analyze binary variables, Fisher’s exact test was used. No adjustment for multiple testing was done. A two-sided *p* value < 0.05 was interpreted as significant. Because of the nature of the study, all statistical test results should be interpreted as explorative.

## Results

### Subject and treatment characteristics

From July 2017 to May 2020, a total of 53 adult SMA patients were treated with nusinersen (defined as at least one successful injection). Among these, two patients had SMA type 1, 15 patients had SMA type 2, and 36 patients had SMA type 3. SMA type 1 patients were not considered in the statistical analysis because of their small number and the limited set of data. Furthermore, one female SMA type 2 patient was excluded from analyses because the acquisition of CSF and administrations of nusinersen were performed using a port system with a spinal catheter. For detailed subject and treatment data, see Table 1.

**Table 1:** Subject and treatment characteristics

	SMA type 2	SMA type 3	Total
Number of patients	14	36	50
Male	5 (35.7%)	26 (72.2%)	31 (62.0%)
Female	9 (64.3%)	10 (27.8%)	19 (38.0%)
Ambulatory	0 (0%)	17 (47.2%)	17 (34.0%)
Spondylodesis	7 (50.0%)	6 (16.7%)	13 (26.0%)
Age in years (mean ± SD)	32.3 ± 10.9	37.8 ± 12.9	36.3 ± 12.5
Age in years (range)	18–52	18–71	18–71
HFMSE baseline score (mean ± SD)	2.4 ± 2.2	28.2 ± 21.2	21.0 ± 21.4
RULM baseline score (mean ± SD)	8.6 ± 5.5	25.7 ± 12.1	20.9 ± 13.2
Injection mode, Number of patients	14	36	50
Conventional	0 (0%)	23 (63.9%)	23 (46.0%)
Fluoroscopy	0 (0%)	4 (11.1%)	4 (8.0%)
CT	14 (100%)	9 (25.0%)	23 (46.0%)
Injection mode, Number of injections	114	290	404
Conventional	0 (0%)	195 (67.2%)	195 (48.3%)
Fluoroscopy	0 (0%)	25 (8.6%)	25 (6.2%)
CT	114 (100%)	70 (24.1%)	184 (45.5%)
Treatment duration			
Patients with 12 injections = 34 months	0	2	2
Patients with 11 injections = 30 months	1	8	9
Patients with 10 injections = 26 months	3	4	7
Patients with 9 injections = 22 months	3	3	6
Patients with 8 injections = 18 months	3	1	4
Patients with 7 injections = 14 months	2	5	7
Patients with 6 injections = 10 months	1	5	6
Patients with 5 injections = 6 months	0	6	6
Patients with 4 injections = 2 months	0	2	2
Patients with 3 injections = 1 month	0	0	0
Patients with 2 injections = 14 days	1	0	1
Patients with 1 injection = baseline	0	0	0
Total observation period (patient-years)	22.2	54.7	76.9

*SMA* spinal muscular atrophy, *SD* standard deviation, *HFMSE* Hammersmith Functional Motor Scale Expanded, *RULM* Revised Upper Limb Module, *CT* computed tomography

### Sample characteristics

A full CSF data set (i.e., CSF-WBC count, total protein, glucose and lactate) was available from 50 of 50 baseline punctures (100%) and from 391 of 404 punctures performed in total (96.8%). In ten cases (2.5%), CSF analyses were not feasible, which was due to insufficient sample volume. In two cases (0.5%), CSF analyses included only the WBC and RBC counts. Conversely, in one case (0.2%), the cell count was missing. A total of 31 of 393 available CSF samples (7.9%, including three baseline samples) from 22 patients were excluded from statistical evaluation because of red blood cell contamination; thus, a total of 362 samples remained for further evaluation.

With regard to blood analyses, baseline values were available with the following frequencies: blood count (WBC and platelets) in 48 cases (96.0%), kidney values (creatinine and urea nitrogen) and liver values (ALT, AST, and GGT) in 46 cases each (92.0%), and CRP in 44 cases (88.0%). Coagulation parameters (aPTT and INR) were found in 28 cases (56.0%), as they were usually collected from hospitalized patients. The total number of available measurements was 327 for blood counts (80.9%), 322 (urea nitrogen) and 323 (creatinine) for kidney values (79.7% and 80.0%, respectively), and 321 each for liver values and CRP (79.5%). Coagulation parameters were available in 180 (aPTT) and 181 (INR) cases (44.6% and 44.8%, respectively).

### Laboratory analyses—baseline values and changes during treatment with nusinersen

Detailed results of baseline parameters (therapy-naïve patients = L1), including subgroup comparisons between SMA type 2 and type 3, are provided in Table 2 and in the Supplemental Materials (Tables S1a–m). The results of the following injections (L2 to M8) are shown in Tables S1a–m. Mean changes from baseline for the total cohort and subgroups, including corresponding observation periods, are presented in Tables 3 and S2a–m. The number of shifts to abnormal laboratory values in individual patients is shown in Table 4.

**Table 2:** Baseline values with subgroup comparison (SMA type 2 versus SMA type 3)

	SMA	n_BL_	n_BL_↑	n_BL_↓	Mean^a^ ± SD^a^	Range^a^	*p* value^b^
CSF							
WBC (/µL)	2	13^c^	1	n/a	2.3 ± 4.3	0–16	
	3	34^c^	2	n/a	2.4 ± 2.4	0–13	
	Total	47^c^	3	n/a	2.4 ± 3.0	0–16	0.15
Glucose (mg/dL)	2	13^c^	n/a	0	62.5 ± 5.8	53–72	
	3	34^c^	n/a	0	65.7 ± 5.4	56–75	
	Total	47^c^	n/a	0	64.8 ± 5.7	53–75	0.12
Lactate (mmol/L)	2	13^c^	0	n/a	1.35 ± 0.19	1.0–1.7	
	3	34^c^	1	n/a	1.57 ± 0.27	1.1–2.5	
	Total	47^c^	1	n/a	1.51 ± 0.27	1.0–2.5	0.01
Protein (mg/dL)	2	13^c^	1	0	34.1 ± 8.6	19–46	
	3	34^c^	11	0	41.9 ± 12.4	26–72	
	Total	47^c^	12	0	39.7 ± 11.9	19–72	0.07
Blood							
WBC (/nL)	2	13	2	0	7.2 ± 2.2	3.9–11.7	
	3	35	5	0	7.1 ± 1.8	4.6–11.9	
	Total	48	7	0	7.1 ± 1.9	3.9–11.9	0.94
CRP (mg/dL)	2	13	5	n/a	–	< 0.5–1.5	
	3	31	10	n/a	–	< 0.5–2.3	
	Total	44	15	n/a	–	< 0.5–2.3	0.68
Platelets (/nL)	2	13	5	2	315.2 ± 122.0	103–477	
	3	35	0	0	265.9 ± 50.9	176–368	
	Total	48	5	2	279.3 ± 78.5	103–477	0.07
INR	2	12	0	n/a	1.02 ± 0.06	0.92–1.12	
	3	16	1	n/a	1.02 ± 0.08	0.93–1.19	
	Total	28	1	n/a	1.02 ± 0.07	0.92–1.19	0.96
aPTT (s)	2	12	0	n/a	28.8 ± 2.6	24.3–32.1	
	3	16	1	n/a	27.5 ± 3.1	20.9–32.9	
	Total	28	1	n/a	28.1 ± 2.9	20.9–32.9	0.29
Crea (mg/dL)	2	13	0	13	0.31 ± 0.05	0.26–0.43	
	3	33	0	24	0.49 ± 0.14	0.28–0.78	
	Total	46	0	37	0.44 ± 0.15	0.26–0.78	0.0001
Urea (mg/dL)	2	13	0	2	8.8 ± 4.1	4.0–18.0	
	3	33	3	1	11.9 ± 4.3	5.0–22.0	
	Total	46	3	3	11.0 ± 4.5	4.0–22.0	0.02
AST (U/L)	2	13	0	n/a	21.0 ± 8.9	11–39	
	3	33	1	n/a	28.5 ± 13.1	11–72	
	Total	46	1	n/a	26.4 ± 12.4	11–72	0.07
ALT (U/L)	2	13	2	n/a	31.5 ± 29.1	8–111	
	3	33	14	n/a	47.7 ± 24.2	17–118	
	Total	46	16	n/a	43.2 ± 26.4	8–118	0.01
GGT (U/L)	2	13	2	n/a	36.1 ± 45.1	7–176	
	3	33	7	n/a	43.7 ± 39.3	7–190	
	Total	46	9	n/a	41.5 ± 40.7	7–190	0.14

*CSF* cerebrospinal fluid, *SMA* spinal muscular atrophy, *WBC* white blood cell count, *CRP* C-reactive protein, *INR* international normalized ratio, *aPTT* activated partial thromboplastin time, *crea* creatinine, *urea* urea nitrogen, *AST* aspartate aminotransferase, *ALT* alanine aminotransferase, *GGT* gamma-glutamyltransferase, *n*_*BL*_ number of available baseline values, *n*_*BL*_*↑* number of elevated baseline values, *n*_*BL*_*↓* number of lowered baseline values, *SD* standard deviation, *n/a* not applicable (only medically reasonable or meaningful directions of deviation were considered)

^a^Units as specified in the first column

^b^SMA type 2 versus SMA type 3

^c^Three CSF baseline samples were excluded from statistical analyses because of red blood cell contamination

**Table 3:** Mean change from baseline under therapy (last available versus baseline values)

	SMA	*N*	Observ. period Mean (months)	Baseline Mean^a^	Change^b^ Mean^a^	*p* value
CSF						
WBC (/µL)	2	12	20.7	2.4	+ 0.2	0.51
	3	34	17.8	2.4	− 0.4	0.50
	Total	46	18.5	2.4	− 0.2	1.00
Glucose (mg/dL)	2	12	20.7	62.6	− 1.7	0.75
	3	34	17.6	65.7	− 2.1	0.04
	Total	46	18.4	64.9	− 2.0	0.04
Lactate (mmol/L)	2	12	20.7	1.33	+ 0.10	0.18
	3	34	17.6	1.57	+ 0.01	0.69
	Total	46	18.4	1.51	+ 0.03	0.23
Protein (mg/dL)	2	12	20.7	33.2	+ 3.8	0.75
	3	34	17.6	41.9	+ 2.6	0.01
	Total	46	18.4	39.6	+ 2.9	0.01
Blood						
WBC (/nL)	2	13	17.3	7.2	+ 0.1	0.58
	3	35	17.4	7.1	− 0.2	0.74
	Total	48	17.4	7.1	− 0.1	1.00
Platelets (/nL)	2	13	17.3	315.2	− 39.0	0.27
	3	35	17.4	265.9	+ 2.7	0.86
	Total	48	17.4	279.3	− 8.6	0.77
INR	2	12	18.2	1.02	+ 0.01	0.75
	3	13	12.0	1.03	+ 0.01	1.00
	Total	25	15.0	1.02	+ 0.01	0.83
aPTT (s)	2	12	18.2	28.8	− 2.1	0.04
	3	13	12.0	28.1	− 0.7	0.09
	Total	25	15.0	28.4	− 1.4	0.004
Crea (mg/dL)	2	13	16.7	0.31	− 0.04	0.15
	3	33	17.9	0.49	− 0.11	< 0.0001
	Total	46	17.5	0.44	− 0.09	< 0.0001
Urea (mg/dL)	2	13	16.7	8.8	+ 1.5	0.27
	3	33	17.8	11.9	− 0.5	0.58
	Total	46	17.4	11.0	+ 0.0	1.00
AST (U/L)	2	13	16.3	21.0	− 1.6	0.09
	3	33	17.8	28.5	+ 1.2	1.00
	Total	46	17.4	26.4	+ 0.4	0.46
ALT (U/L)	2	13	16.3	31.5	− 4.7	1.00
	3	33	17.8	47.7	− 1.6	0.22
	Total	46	17.4	43.2	− 2.5	0.36
GGT (U/L)	2	13	16.3	36.1	+ 3.5	1.00
	3	33	17.8	43.7	− 6.2	0.49
	Total	46	17.4	41.5	− 3.5	0.46

*CSF* cerebrospinal fluid, *SMA* spinal muscular atrophy,* WBC* white blood cell count, *INR* international normalized ratio, *aPTT* activated partial thromboplastin time, *crea* creatinine, *urea* urea nitrogen, *AST* aspartate aminotransferase, *ALT* alanine aminotransferase, *GGT* gamma-glutamyltransferase, *n* number of patients with at least one baseline and one follow-up sample, *observ. period* observation period

^a^Units as specified in the first column

^b^For each patient, the last measured value was compared with the respective baseline value

**Table 4:** Number of patients with shifts to abnormal laboratory values under therapy

	Baseline	Deviation from normal range under therapy				
	Normal	Dir	Sporadic^a^	Transient^b^	Persistent^c^	No deviation
CSF						
WBC	44	**⬈** **⬊**	7 (15.9%) n/a	2 (4.5%) n/a	0 (0.0%) n/a	35 (79.5%)
Glucose	47	**⬈** **⬊**	n/a 0 (0.0%)	n/a 0 (0.0%)	n/a 0 (0.0%)	47 (100%)
Lactate	46	**⬈** **⬊**	3 (6.5%) n/a	1 (2.2%) n/a	0 (0.0%) n/a	42 (91.3%)
Protein	35	**⬈** **⬊**	6 (17.1%) n/a	3 (8.6%) n/a	2 (5.7%) n/a	24 (68.6%)
Blood						
WBC	41	**⬈** **⬊**	7 (17.1%) 1 (2.4%)	0 (0.0%) 0 (0.0%)	1 (2.4%) 0 (0.0%)	32 (78.0%)
CRP	29	**⬈** **⬊**	9 (31.0%) n/a	3 (10.3%) n/a	0 (0.0%) n/a	17 (58.6%)
Platelets	41	**⬈** **⬊**	1 (2.4%) 1 (2.4%)	1 (2.4%) 0 (0.0%)	0 (0.0%) 0 (0.0%)	38 (92.7%)
INR	27	**⬈** **⬊**	2 (7.4%) n/a	0 (0.0%) n/a	0 (0.0%) n/a	25 (92.6%)
aPTT	27	**⬈** **⬊**	4 (14.8%) n/a	0 (0.0%) n/a	0 (0.0%) n/a	23 (85.2%)
Crea	9	**⬈** **⬊**	0 (0.0%) 2 (22.2%)	0 (0.0%) 3 (33.3%)	0 (0.0%) 1 (11.1%)	3 (33.3%)
Urea	40	**⬈** **⬊**	3 (7.5%) 3 (7.5%)	0 (0.0%) 0 (0.0%)	0 (0.0%) 0 (0.0%)	34 (85.0%)
AST	45	**⬈** **⬊**	2 (4.4%) n/a	1 (2.2%) n/a	1 (2.2%) n/a	41 (91.1%)
ALT	30	**⬈** **⬊**	2 (6.7%) n/a	1 (3.3%) n/a	0 (0.0%) n/a	27 (90.0%)
GGT	37	**⬈** **⬊**	0 (0.0%) n/a	2 (5.4%) n/a	1 (2.7%) n/a	34 (91.9%)

*CSF* cerebrospinal fluid, *WBC* white blood cell count, *CRP* C-reactive protein, *INR* international normalized ratio, *aPTT* activated partial thromboplastin time, *crea* creatinine, *urea* urea nitrogen, *AST* aspartate aminotransferase, *ALT* alanine aminotransferase, *GGT* gamma-glutamyltransferase, *Dir* direction, ⬈, increased/elevated, ⬊ decreased/lowered, *n/a* not applicable (only medically reasonable or meaningful directions of deviation were considered)

^a^Only singular deviations from the normal range with immediate normalization afterwards

^b^Two or more deviations in a row with normalization afterwards or two deviations in a row at the end of the observation period

^c^Three or more deviations in a row without normalization until the end of the observation period

#### CSF parameters

At baseline, the mean CSF-WBC count was 2.4/µL, with no difference between SMA type 2 and type 3 subgroups. Pleocytosis was seen in three cases (6.4%). One SMA type 2 patient with spondylodesis, a restrictive ventilation disorder with documented recurrent pneumonias, and noninvasive ventilation at night showed pleocytosis of 16/µL (14/µL mononuclear cells, 2/µL granulocytes). In another patient with SMA type 3 and spondylodesis, the CSF-WBC count was 13/µL, and all of them were granulocytes. The third case was an SMA type 3 patient with a CSF-WBC count of 6/µL (5/µL mononuclear cells, 1/µL granulocytes). However, none of these patients had any clinical evidence of infectious or noninfectious CNS inflammation. Under therapy with nusinersen, the mean CSF-WBC count remained stable over time. However, during the first three injections, there was a trend towards higher CSF-WBC counts (*p* = 0.05). From 44 subjects with normal CSF-WBC counts at baseline, nine showed sporadic or transient elevations up to 21/µL during the observation. No patient developed persistent pleocytosis.

Glucose levels at baseline were normal in all patients, with no subgroup differences. Mean glucose levels decreased by 2 mg/dL (*p* = 0.04). Since CSF glucose levels are associated with blood glucose levels, this slight change was not considered clinically relevant. In addition, no shifts to abnormal glucose levels were observed during therapy.

Mean lactate levels at baseline were normal but lower in SMA type 2 patients than in SMA type 3 patients (*p* = 0.01). In a single SMA type 3 patient, lactate was slightly elevated to 2.5 mmol/L at the first injection. In the same patient, total protein was elevated to 68 mg/dL, indicating a BBB disturbance, whereas CSF-WBC count and glucose were normal. In general, mean lactate levels remained stable during the course of therapy. Three patients showed a sporadic elevation of lactate, while one patient had slightly elevated lactate levels up to 2.3 mmol/L in several samples, two of them consecutively. Analogous to the case mentioned above, this patient already showed a high-normal baseline value of 2.1 mmol/L and elevated total protein levels, probably indicating a BBB disorder.

In therapy-naïve patients, total protein was in the upper normal range with a mean value of 39.7 mg/dL and a trend towards higher levels in SMA type 3 patients (*p* = 0.07). Normal values were obtained from 35 patients. In eleven SMA type 3 patients, elevations up to 72 mg/dL were observed before therapy started. In contrast, there was only one SMA type 2 patient with a marginal elevation to 46 mg/dL. Under therapy, the total cohort showed an increase to 42.5 mg/dL (*p* = 0.01). In the subgroup analysis, total protein increased by 3.8 mg/dL in SMA type 2 and 2.6 mg/dL in SMA type 3 patients. However, this difference was significant only in SMA type 3 (*p* = 0.01). Nine patients with normal baseline values showed sporadic or transient increases up to 76 mg/dL during therapy. A persistent increase was observed in two patients (5.7%): One SMA type 3 patient with a high-normal baseline of 43 mg/dL developed values up to 61 mg/dL from M3 to M6 (end of the observation period). In addition, a single SMA type 2 patient with a baseline value of 36 mg/dL developed elevated total protein levels up to 67 mg/dL from M2 to M5 (end of observation). In both patients, the other CSF parameters showed no alterations.

#### Inflammatory markers

The WBC count was elevated in 7 of 48 available baseline samples with a maximum of 11.9/nL. The CRP was slightly elevated in 15 of 44 baseline samples with a maximum of 2.3 mg/dL. For both parameters, there were no differences between SMA type 2 and SMA type 3 patients. Mean WBC counts remained stable under therapy with nusinersen. Most subjects showed no or only slight sporadic increases in the WBC count up to 11.9/nL. A single SMA type 2 patient (2.4%) showed mild leukocytosis up to 11.1/nL in the last three available measurements. In the same patient, CRP levels were slightly increased up to 0.8 mg/dL, possibly indicating mild systemic inflammation.

#### Platelets

At baseline, mean platelet levels were normal, and platelet count abnormalities were only observed in SMA type 2 patients. The platelet count was lowered in two patients with a minimum value of 103/nL, whereas thrombocytosis was observed in five subjects with a maximum of 477/nL. During therapy with nusinersen, there was no evidence of a change in the mean platelet count, either between the first and last injections or during the loading dosing. Analyses of individual patients revealed only a few shifts to abnormal values. Of 41 patients with normal platelet levels at baseline, a single SMA type 2 patient showed sporadic mild thrombocytopenia of 101/nL at M4, although dilution of the blood count cannot be ruled out, as leukocytes were also lower than usual. Furthermore, one SMA type 3 patient showed transient thrombocytosis up to 532/nL at M1/M2 with subsequent normalization (M3-M6). Another SMA type 3 patient showed singular thrombocytosis of 422/nL at M1.

#### Coagulation parameters

Baseline coagulation values were normal in most patients. Only one SMA type 3 patient showed a marginal prolonged aPTT to 32.9 s, whereas another SMA type 3 patient showed a slight increase in INR to 1.19. No change of mean INR was observed over time. In contrast, the mean aPTT decreased by 1.4 s in the total cohort (*p* = 0.004) and 2.1 s in SMA type 2 patients (*p* = 0.04). During the observation period, there were only a few and slight sporadic increases in aPTT and INR to a maximum of 33.3 s and 1.18, respectively.

#### Kidney values

In therapy-naïve patients, creatinine levels were lowered in all SMA type 2 patients and in 24 of 33 SMA type 3 patients. Remarkably, the mean values of both subgroups were below the normal range (0.31 and 0.49 mg/dL, respectively). Furthermore, SMA type 2 patients had significantly lower creatinine values than SMA type 3 patients (*p* = 0.0001). Mean urea nitrogen levels were normal, with higher levels found in SMA type 3 patients (*p* = 0.02). In three patients each, urea nitrogen was slightly decreased or increased. During the observation period, the mean creatinine levels decreased by 0.09 mg/dL in the total cohort (*p* < 0.0001), while a decrease of 0.11 mg/dL was found in SMA type 3 patients (*p* < 0.0001). In contrast, the mean urea nitrogen levels remained unchanged over time. Of nine SMA type 3 patients with normal baseline values, six developed lowered creatinine levels during the course of therapy, with one decrease being persistent (11.1%). Urea nitrogen levels changed only sporadically and mildly in a few cases, with a minimum of 5 mg/dL and a maximum of 23 mg/dL.

#### Liver values

At baseline, mean AST levels were normal, with SMA type 3 patients tending to have higher values than type 2 patients (*p* = 0.07). An elevated AST to 72 U/L was observed in a single SMA type 3 patient. In comparison, ALT and GGT were more frequently elevated (16 and 9 cases with a maximum of 118 and 190 U/L, respectively). Subgroup comparison showed higher ALT levels in SMA type 3 patients (*p* = 0.01), whereas for GGT, there was no difference between SMA types. Under therapy, analysis of mean enzyme levels revealed no relevant changes in either the total cohort or the SMA subgroups. In individual patients, few and mainly sporadic or transient elevations were observed. However, one SMA type 3 patient (2.2%) developed mild but persistent elevated AST values up to 58 U/L, while one SMA type 2 patient (2.7%) showed a successive and persistent increase in GGT up to 101 U/L from M3 to M5 (end of observation).

#### Influence of the injection method on laboratory values

A possible relationship between the injection method (conventional vs. image-guided) and the change in laboratory values from baseline was investigated. In summary, there was no evidence that the injection method had any influence on the development of laboratory values under therapy (data not shown).

## Discussion

Laboratory parameters can help to identify side effects and organ toxicities of new therapies and thus to provide information on therapy safety. In the past, a variety of side effects have been identified for different ASOs, such as platelet declines, renal impairment, increases in liver enzymes or prolongation of aPTT [3, 7, 15, 20]. In particular, thrombocytopenia, including acute severe thrombocytopenia, and nephrotoxicity are major concerns and are often considered class effects. However, these side effects seem to occur only with the use of some ASOs [8–10, 15]. Since data on laboratory findings in adult SMA patients under therapy with nusinersen are lacking, the present study helps to assess the safety of the therapy better.

Analysis of CSF parameters can detect various pathologies, such as infectious or noninfectious CNS inflammation, intrathecal hemorrhage, changes in CSF flow, or BBB dysfunction. Changes in CSF parameters under therapy with nusinersen are of particular interest since the drug has to be administered by repeated lumbar punctures. In our cohort, the mean baseline values of the analyzed CSF parameters were all within the normal range. Three patients showed mild pleocytosis at baseline; however, an association with SMA is unlikely. In addition, the mean CSF-WBC count tended to increase during the first three injections (L1–L3). Furthermore, approximately half of the CSF samples with pleocytosis were obtained at L2 or L3. In line with our results, Müschen et al. and Wurster et al. found a transient increase in mean CSF-WBC counts during loading dosing. Also in these studies, all increases in CSF-WBC counts (four increases in three of 88 SMA patients) occurred at L2 or L3 [32, 41]. These observations are most likely a reaction to the rapid succession of lumbar punctures at the beginning of the therapy. Under therapy, we found mild sporadic or transient elevations in CSF-WBC counts in approximately one-fifth of the patients, whereas no persistent increase was seen. In contrast, Müschen et al., Wurster et al. and Kessler et al. observed only three cases of pleocytosis in 28, 60 and 10 SMA patients treated for a period of 2 to 18 months [21, 32, 41].

Total CSF protein, an indicator of BBB and CSF flow integrity, was elevated in eleven SMA type 3 patients but only in one SMA type 2 patient. However, the widely used upper limit of normal (ULN) of 45 mg/dL is now being challenged [5, 6]. Although significance was not reached in our cohort, the trend of higher CSF protein concentrations in SMA type 3 patients is consistent with the findings of Müschen et al. and Wurster et al. [32, 41]. Since total protein increases with age, the difference in patient age between the SMA type 2 and type 3 subgroups could be an explanation although it was only 5.5 years in our cohort [27]. During nusinersen therapy, two patients in our cohort developed a sustained elevation of total protein. Furthermore, mean protein levels increased significantly in the total cohort as well as in the SMA type 3 subgroup. These findings are compatible with results from other studies on nusinersen in adult patients and may indicate an alteration of CSF flow or BBB dysfunction. Whether this observation is a consequence of repeated lumbar punctures or a side effect of the drug remains unclear [21, 32, 39, 41]. This outcome may possibly be related to reports of rare cases of hydrocephalus of unknown etiology.

Recently, some cases of severe thrombocytopenia with platelet counts < 25/nL have been reported in clinical trials on ASOs. In the NEURO-TTR trial, three cases (3%) of severe thrombocytopenia, including one in association with a fatal intracranial hemorrhage, were observed during therapy with inotersen, an ASO designed to inhibit hepatic production of transthyretin in patients with hereditary transthyretin amyloidosis. In the same study, a decrease in platelet count to less than 140/nL was seen in 54% of the participants in the inotersen group vs. 13% in the placebo group [4]. In another study, severe thrombocytopenia was found in 2 of 33 patients with familial chylomicronemia treated with volanesorsen [40]. Based on experience with other ASOs, thrombocytopenia, including acute severe thrombocytopenia, is part of the warnings in the prescribing information of nusinersen. Data on platelet counts under therapy with nusinersen are mainly available from studies in infants or children. In the ENDEAR and CHERISH studies, the incidence of shifts to low platelet counts was 13 vs. 0% (nusinersen vs. placebo) and 20 vs. 26%, respectively. Platelet counts < 20/µL were seen in one infant in the ENDEAR study and in two children in the CHERISH study. However, no sustained shifts or bleeding complications occurred. Median platelet counts remained stable during therapy [11]. Another study of nusinersen therapy in presymptomatic infants also showed stable platelet counts [12]. In a cohort of 58 SMA patients aged between 1 month and 56 years, Goedeker et al. described three cases of mild thrombocytopenia, with none of them reaching a platelet count < 100/nL[17]. Szabo et al. reported singular mild and transient thrombocytopenia in a single child treated with nusinersen [38]. Remarkably, only a single case of sporadic thrombocytopenia with a platelet count of 101/nL occurred in our study. For the graduation of abnormal laboratory values, National Cancer Institute—Common Terminology Criteria of Adverse Events (NCI-CTCAE version 5.0) is often used in clinical studies, which distinguishes between four severity grades (grade 1, mild, to grade 4, severe) [1]. According to this reference, the observed thrombocytopenia would meet the criterion of grade 1 thrombocytopenia, defined as a platelet count between the lower limit of normal (LLN) and 75/nL. Similar to the data from pediatric studies, platelet levels remained stable over time. Based on our results, there is currently no evidence of an increased risk of severe thrombocytopenia in adult patients treated with nusinersen.

We observed a reduction in the mean aPTT between the first and last injections in the total cohort and the SMA type 2 subgroup. Shortened aPTTs are often considered laboratory artifacts, which are due to difficult blood sampling. In addition, there is some evidence that short aPTTs represent a hypercoagulable state and may be associated with an increased risk for thromboembolic and cardiovascular events [23, 30]. With regard to the coagulation parameters, we did not observe any persistent parameter deviations from the normal range, which is in line with the results of Goedeker et al. [17].

Nephrotoxicity is a major concern with the use of ASOs [3, 15]. A significantly increased risk of renal impairment was observed in patients with Duchenne muscular dystrophy treated with drisapersen [36]. In the abovementioned study on inotersen, three cases of glomerulonephritis and one case of tubulointerstitial nephritis were observed in 112 subjects with hereditary transthyretin amyloidosis [4]. In pivotal studies on nusinersen, shifts to high urea nitrogen or creatinine were observed only in individual cases. Additionally, proteinuria did not occur more frequently in nusinersen-treated subjects than in control subjects, so the studies did not provide evidence of renal impairment [11, 14, 29]. In the study of Goedeker et al. proteinuria was the most common laboratory abnormality. However, it was never persistent and often attributed to sample contamination [17]. A safety evaluation of 18 adult patients with SMA type 3 and type 4 treated with nusinersen over a 14-month period revealed no evidence of renal dysfunction during therapy [13]. In our cohort, mean creatinine levels at baseline were decreased in both the SMA type 2 and SMA type 3 subgroups, with SMA type 2 patients having lower values than SMA type 3 patients (0.31 vs. 0.49 mg/dL). These observations are not surprising since creatinine levels correlate with skeletal muscle mass and are consistent with a study conducted in 238 patients with SMA types 1–3. In line with our results, the authors found 1.7-fold higher creatinine levels in SMA type 3 than in SMA type 2. Furthermore, serum creatinine correlated with SMA type, *SMN2* gene copy number, motor function, and severity of denervation. The authors conclude that creatinine is a candidate biomarker for SMA progression and suggest investigating whether creatinine levels respond to new SMA therapies [2, 22]. Remarkably, only nine SMA type 3 patients from our cohort had normal creatinine values at baseline, and six of them dropped below the LLN during the course of therapy, with one decrease classified as persistent. Furthermore, mean creatinine levels decreased in the SMA type 3 subgroup and the total cohort, possibly indicating further disease progression under therapy. With regard to nephrotoxicity, we found no case of a relevant increase in either serum creatinine or urea nitrogen. However, the use of serum creatinine as a marker of renal function is strongly limited in SMA patients for the reason already mentioned above. Therefore, determination of cystatin C protein might be a more appropriate method for monitoring renal function in patients with SMA [13, 22].

Hepatotoxicity or an increase in liver enzymes are other potential side effects with the use of ASOs [15]. In clinical trials, mipomersen, an ASO used to treat dyslipidemia, resulted in an increased incidence of elevated ALT above 3 × ULN and hepatic steatosis compared to placebo [33]. With regard to nusinersen, the NURTURE trial showed stable transaminases in presymptomatic children [12]. In ENDEAR and CHERISH studies, there were some shifts in ALT and AST, with no clear difference from the control groups or reports of liver failures [11, 14, 29]. Similar to the results from pediatric studies, only a few and mostly temporary elevations in liver enzymes were observed in our cohort. However, one patient with SMA type 2 with a normal baseline value developed a discrete but persistent increase in AST, while a single SMA type 2 patient showed a successive increase in GGT. Remarkably, both cases would only fulfill grade 1 according to the NCI-CTCAE, defined as enzyme levels up to 3 × ULN [1]. Regardless of the baseline value, no patient in our study developed elevations in liver enzymes above threefold the respective baseline value, and no patient with elevated liver enzymes showed increased bilirubin levels (data not shown). In conclusion, there was no evidence of a clinically relevant increase under treatment with nusinersen.

In terms of detecting potential toxicities of nusinersen therapy in adult patients, this study has some limitations. Importantly, despite the relatively long observation period, rare side effects may not have been captured because of the limited cohort size. In outpatients, coagulation parameters were not usually assessed during therapy. Thus, a large proportion of samples and results were from more severely affected patients, which may result in a bias. The other blood samples from outpatients were usually acquired before the first loading dose and each maintenance dose. Therefore, transient changes during loading dosing in these patients may have been missed. Finally, our definition of the duration of deviations from the normal range is somewhat arbitrary. In particular, "persistent" changes may have returned to normal values after the end of the observation period, and conversely, "transient" shifts may have persisted.

## Conclusion

Our data demonstrate a favorable safety profile of nusinersen therapy in adult SMA patients under longer-term “real-world” conditions, regardless of SMA type. In particular, we found no evidence of clinically relevant platelet declines, coagulopathies, or renal or hepatic organ toxicities, which are common concerns with the use of ASOs. Whether this finding is solely attributable to the intrathecal administration route of nusinersen remains speculative.

## Supplementary Information

Below is the link to the electronic supplementary material.

Supplementary file1 (PDF 2046 KB)

## Data Availability

The data that support the findings of this study are available on request from the corresponding author. The data are not publicly available due to privacy or ethical restrictions.
