# Supplementary material for: Nusinersen treatment in adult patients with spinal muscular atrophy: a safety analysis of laboratory parameters
Source: J Neurol. 2021 Apr 25;268(12):4667–79. doi: 10.1007/s00415-021-10569-8 (PMC8563549; doi:10.1007/s00415-021-10569-8)
Supplement: Supplementary file 1 — Supplementary file1 (PDF 2046 KB) [file 415_2021_10569_MOESM1_ESM.pdf]

Supplemental Materials

Nusinersen Treatment in Adult Patients with Spinal Muscular Atrophy - A Safety Analysis of Laboratory Parameters

Journal of Neurology

Benjamin Stolte[1,5], Michael Nonnemacher[2], Kathrin Kizina[1,5], Saskia Bolz[1,5], Andreas Totzeck[1,5], Andreas Thimm[1,5], Bernd Wagner[3], Cornelius Deuschl[4,5], Christoph Kleinschnitz[1,5], Tim Hagenacker[1,5]

[1]Department of Neurology, University Hospital Essen, Essen, Germany
[2]Institute for Medical Informatics, Biometrics and Epidemiology, University Hospital Essen, Essen, Germany
[3]Department of Clinical Chemistry, University Hospital Essen, Essen, Germany
[4]Institute for Diagnostic and Interventional Radiology and Neuroradiology, University Hospital Essen, Essen, Germany
[5]Center for Translational and Behavioral Neuroscience, University Hospital Essen, Essen, Germany

Corresponding author:
Tim Hagenacker, MD
Department of Neurology
University Hospital Essen
Hufelandstr. 55
45147 Essen
Germany
e-mail: tim.hagenacker@uk-essen.de
phone: +49 201 723 6513

Table S1a. Descriptive statistics: Cerebrospinal fluid (CSF) - White blood cell (WBC) count [number/µL].

SMA type	Injection	N	Mean	Std Dev	Median	Lower Quartile	Upper Quartile	Quartile Range	Minimum	Maximum
SMA type 2	L1	13	2.308	4.250	1.000	1.000	2.000	1.000	0.000	16.000
	L2	9	3.000	4.848	1.000	0.000	4.000	4.000	0.000	15.000
	L3	13	3.846	4.913	1.000	0.000	5.000	5.000	0.000	13.000
	L4	11	1.909	3.145	1.000	0.000	2.000	2.000	0.000	11.000
	M1	11	2.455	5.556	1.000	0.000	1.000	1.000	0.000	19.000
	M2	9	1.222	0.972	1.000	1.000	2.000	1.000	0.000	3.000
	M3	12	2.083	3.728	1.000	0.000	2.000	2.000	0.000	13.000
	M4	8	0.875	1.356	0.500	0.000	1.000	1.000	0.000	4.000
	M5	7	1.429	1.272	1.000	1.000	2.000	1.000	0.000	4.000
	M6	3	1.667	1.528	2.000	0.000	3.000	3.000	0.000	3.000
	M7	1	1.000	.	1.000	1.000	1.000	0.000	1.000	1.000
	M8	0	.	.	.	.	.	.	.	.
SMA type 3	L1	34	2.441	2.439	1.500	1.000	3.000	2.000	0.000	13.000
	L2	33	2.909	2.529	3.000	1.000	4.000	3.000	0.000	12.000
	L3	32	2.844	1.903	2.500	1.000	3.000	2.000	0.000	7.000
	L4	35	2.257	2.105	2.000	1.000	3.000	2.000	0.000	11.000
	M1	31	2.032	1.560	2.000	1.000	3.000	2.000	0.000	6.000
	M2	26	1.962	1.371	2.000	1.000	2.000	1.000	0.000	7.000
	M3	19	2.842	4.549	2.000	1.000	3.000	2.000	0.000	21.000
	M4	17	2.471	1.419	2.000	1.000	3.000	2.000	1.000	6.000
	M5	15	2.067	1.668	2.000	1.000	3.000	2.000	0.000	7.000
	M6	13	2.385	2.434	2.000	1.000	2.000	1.000	1.000	10.000
	M7	8	2.500	1.690	2.000	1.000	4.000	3.000	1.000	5.000
	M8	2	2.500	0.707	2.500	2.000	3.000	1.000	2.000	3.000
Total	L1	47	2.404	2.998	1.000	1.000	3.000	2.000	0.000	16.000
	L2	42	2.929	3.095	2.000	1.000	4.000	3.000	0.000	15.000
	L3	45	3.133	3.057	2.000	1.000	4.000	3.000	0.000	13.000
	L4	46	2.174	2.360	2.000	1.000	3.000	2.000	0.000	11.000
	M1	42	2.143	3.057	1.000	1.000	2.000	1.000	0.000	19.000
	M2	35	1.771	1.308	2.000	1.000	2.000	1.000	0.000	7.000
	M3	31	2.548	4.202	2.000	0.000	3.000	3.000	0.000	21.000
	M4	25	1.960	1.567	2.000	1.000	3.000	2.000	0.000	6.000
	M5	22	1.864	1.552	2.000	1.000	2.000	1.000	0.000	7.000
	M6	16	2.250	2.266	2.000	1.000	2.000	1.000	0.000	10.000
	M7	9	2.333	1.658	2.000	1.000	3.000	2.000	1.000	5.000
	M8	2	2.500	0.707	2.500	2.000	3.000	1.000	2.000	3.000

Table S1b. Descriptive statistics: Cerebrospinal fluid (CSF) - Glucose [mg/dL].

SMA type	Injection	N	Mean	Std Dev	Median	Lower Quartile	Upper Quartile	Quartile Range	Minimum	Maximum
SMA type 2	L1	13	62.462	5.797	61.000	60.000	67.000	7.000	53.000	72.000
	L2	9	59.889	3.480	59.000	57.000	61.000	4.000	56.000	66.000
	L3	13	62.692	5.721	62.000	59.000	67.000	8.000	53.000	70.000
	L4	11	62.091	6.426	60.000	57.000	69.000	12.000	53.000	71.000
	M1	11	60.182	4.916	60.000	57.000	64.000	7.000	51.000	67.000
	M2	10	60.500	4.927	60.500	57.000	65.000	8.000	54.000	69.000
	M3	12	60.083	4.776	60.500	56.000	64.000	8.000	52.000	66.000
	M4	8	60.125	3.137	59.500	57.500	62.000	4.500	57.000	66.000
	M5	7	59.286	3.946	59.000	57.000	63.000	6.000	53.000	65.000
	M6	3	62.000	7.937	59.000	56.000	71.000	15.000	56.000	71.000
	M7	1	62.000	.	62.000	62.000	62.000	0.000	62.000	62.000
	M8	0	.	.	.	.	.	.	.	.
SMA type 3	L1	34	65.676	5.420	65.500	60.000	70.000	10.000	56.000	75.000
	L2	33	64.909	5.997	64.000	61.000	68.000	7.000	52.000	78.000
	L3	31	64.677	6.274	65.000	59.000	70.000	11.000	55.000	78.000
	L4	35	64.514	4.829	64.000	61.000	69.000	8.000	55.000	74.000
	M1	31	63.710	6.827	62.000	60.000	67.000	7.000	51.000	81.000
	M2	25	62.800	5.888	60.000	58.000	67.000	9.000	56.000	75.000
	M3	19	64.632	7.826	63.000	57.000	71.000	14.000	55.000	84.000
	M4	17	64.176	8.233	62.000	60.000	64.000	4.000	55.000	88.000
	M5	15	63.000	5.593	62.000	60.000	66.000	6.000	53.000	74.000
	M6	13	63.615	6.715	64.000	59.000	68.000	9.000	53.000	74.000
	M7	8	63.375	7.170	64.000	57.500	68.000	10.500	53.000	75.000
	M8	2	62.000	2.828	62.000	60.000	64.000	4.000	60.000	64.000
Total	L1	47	64.787	5.653	64.000	60.000	70.000	10.000	53.000	75.000
	L2	42	63.833	5.897	63.000	59.000	67.000	8.000	52.000	78.000
	L3	44	64.091	6.118	63.000	59.000	69.500	10.500	53.000	78.000
	L4	46	63.935	5.281	64.000	60.000	69.000	9.000	53.000	74.000
	M1	42	62.786	6.517	62.000	59.000	66.000	7.000	51.000	81.000
	M2	35	62.143	5.658	60.000	58.000	66.000	8.000	54.000	75.000
	M3	31	62.871	7.084	63.000	57.000	66.000	9.000	52.000	84.000
	M4	25	62.880	7.196	61.000	58.000	64.000	6.000	55.000	88.000
	M5	22	61.818	5.333	61.000	58.000	64.000	6.000	53.000	74.000
	M6	16	63.313	6.700	63.500	58.000	69.000	11.000	53.000	74.000
	M7	9	63.222	6.723	63.000	58.000	67.000	9.000	53.000	75.000
	M8	2	62.000	2.828	62.000	60.000	64.000	4.000	60.000	64.000

Table S1c. Descriptive statistics: Cerebrospinal fluid (CSF) – Lactate [mmol/L].

SMA type	Injection	N	Mean	Std Dev	Median	Lower Quartile	Upper Quartile	Quartile Range	Minimum	Maximum
SMA type 2	L1	13	1.346	0.185	1.300	1.300	1.500	0.200	1.000	1.700
	L2	9	1.322	0.148	1.300	1.200	1.400	0.200	1.200	1.600
	L3	13	1.423	0.174	1.400	1.300	1.500	0.200	1.200	1.800
	L4	11	1.409	0.176	1.400	1.300	1.500	0.200	1.100	1.700
	M1	11	1.427	0.185	1.400	1.300	1.500	0.200	1.100	1.800
	M2	10	1.280	0.187	1.300	1.100	1.400	0.300	1.000	1.600
	M3	12	1.425	0.201	1.350	1.250	1.600	0.350	1.200	1.700
	M4	8	1.400	0.120	1.400	1.350	1.450	0.100	1.200	1.600
	M5	7	1.471	0.138	1.500	1.300	1.600	0.300	1.300	1.600
	M6	3	1.433	0.058	1.400	1.400	1.500	0.100	1.400	1.500
	M7	1	1.500	.	1.500	1.500	1.500	0.000	1.500	1.500
	M8	0	.	.	.	.	.	.	.	.
SMA type 3	L1	34	1.574	0.265	1.600	1.400	1.700	0.300	1.100	2.500
	L2	33	1.558	0.248	1.600	1.300	1.700	0.400	1.200	2.300
	L3	31	1.629	0.289	1.500	1.400	1.900	0.500	1.000	2.200
	L4	35	1.569	0.261	1.500	1.400	1.800	0.400	1.100	2.100
	M1	31	1.648	0.268	1.600	1.500	1.700	0.200	1.100	2.200
	M2	25	1.552	0.262	1.500	1.400	1.700	0.300	1.100	2.300
	M3	19	1.616	0.269	1.600	1.400	1.800	0.400	1.100	2.100
	M4	17	1.647	0.274	1.700	1.500	1.700	0.200	1.200	2.300
	M5	15	1.627	0.228	1.600	1.500	1.700	0.200	1.200	2.200
	M6	13	1.508	0.193	1.500	1.400	1.500	0.100	1.300	1.900
	M7	8	1.575	0.328	1.500	1.350	1.750	0.400	1.200	2.200
	M8	2	1.450	0.354	1.450	1.200	1.700	0.500	1.200	1.700
Total	L1	47	1.511	0.265	1.500	1.300	1.700	0.400	1.000	2.500
	L2	42	1.507	0.248	1.500	1.300	1.700	0.400	1.200	2.300
	L3	44	1.568	0.275	1.500	1.400	1.800	0.400	1.000	2.200
	L4	46	1.530	0.251	1.500	1.400	1.700	0.300	1.100	2.100
	M1	42	1.590	0.266	1.600	1.500	1.700	0.200	1.100	2.200
	M2	35	1.474	0.270	1.500	1.300	1.600	0.300	1.000	2.300
	M3	31	1.542	0.259	1.600	1.300	1.700	0.400	1.100	2.100
	M4	25	1.568	0.261	1.500	1.400	1.700	0.300	1.200	2.300
	M5	22	1.577	0.214	1.600	1.400	1.700	0.300	1.200	2.200
	M6	16	1.494	0.177	1.450	1.400	1.500	0.100	1.300	1.900
	M7	9	1.567	0.308	1.500	1.400	1.600	0.200	1.200	2.200
	M8	2	1.450	0.354	1.450	1.200	1.700	0.500	1.200	1.700

Table S1d. Descriptive statistics: Cerebrospinal fluid (CSF) – Total protein [mg/dL].

SMA type	Injection	N	Mean	Std Dev	Median	Lower Quartile	Upper Quartile	Quartile Range	Minimum	Maximum
SMA type 2	L1	13	34.077	8.616	35.000	28.000	39.000	11.000	19.000	46.000
	L2	9	36.889	8.283	38.000	35.000	41.000	6.000	24.000	50.000
	L3	13	36.385	11.288	38.000	29.000	41.000	12.000	15.000	54.000
	L4	11	34.455	11.970	32.000	25.000	43.000	18.000	18.000	59.000
	M1	11	36.909	9.944	32.000	29.000	45.000	16.000	24.000	53.000
	M2	10	40.800	13.054	37.500	32.000	50.000	18.000	24.000	67.000
	M3	12	39.583	16.262	41.000	26.500	48.500	22.000	15.000	76.000
	M4	8	38.500	13.784	32.500	30.500	45.500	15.000	24.000	67.000
	M5	7	35.286	12.230	33.000	25.000	45.000	20.000	25.000	58.000
	M6	3	28.667	3.215	30.000	25.000	31.000	6.000	25.000	31.000
	M7	1	26.000	.	26.000	26.000	26.000	0.000	26.000	26.000
	M8	0	.	.	.	.	.	.	.	.
SMA type 3	L1	34	41.882	12.416	39.000	32.000	52.000	20.000	26.000	72.000
	L2	33	43.030	13.660	43.000	32.000	51.000	19.000	23.000	81.000
	L3	31	41.839	13.984	36.000	31.000	54.000	23.000	25.000	80.000
	L4	35	41.743	13.663	38.000	31.000	53.000	22.000	23.000	75.000
	M1	31	41.903	12.931	42.000	31.000	50.000	19.000	23.000	78.000
	M2	25	42.120	14.443	36.000	31.000	51.000	20.000	26.000	74.000
	M3	19	43.737	12.679	41.000	31.000	53.000	22.000	26.000	62.000
	M4	17	46.529	12.420	43.000	37.000	54.000	17.000	29.000	73.000
	M5	15	47.867	12.409	48.000	38.000	61.000	23.000	30.000	69.000
	M6	13	49.615	15.305	51.000	39.000	56.000	17.000	29.000	78.000
	M7	8	48.000	12.705	44.000	37.500	59.500	22.000	34.000	68.000
	M8	2	70.000	7.071	70.000	65.000	75.000	10.000	65.000	75.000
Total	L1	47	39.723	11.934	38.000	32.000	46.000	14.000	19.000	72.000
	L2	42	41.714	12.866	40.000	32.000	50.000	18.000	23.000	81.000
	L3	44	40.227	13.354	37.500	30.000	49.500	19.500	15.000	80.000
	L4	46	40.000	13.519	37.000	30.000	50.000	20.000	18.000	75.000
	M1	42	40.595	12.305	38.500	30.000	49.000	19.000	23.000	78.000
	M2	35	41.743	13.883	36.000	32.000	51.000	19.000	24.000	74.000
	M3	31	42.129	14.059	41.000	29.000	52.000	23.000	15.000	76.000
	M4	25	43.960	13.148	43.000	33.000	52.000	19.000	24.000	73.000
	M5	22	43.864	13.467	42.000	33.000	53.000	20.000	25.000	69.000
	M6	16	45.688	16.127	41.500	30.500	55.500	25.000	25.000	78.000
	M7	9	45.556	13.965	42.000	36.000	58.000	22.000	26.000	68.000
	M8	2	70.000	7.071	70.000	65.000	75.000	10.000	65.000	75.000

Table S1e. Descriptive statistics: White blood cell (WBC) count [number/nL].

SMA type	Injection	N	Mean	Std Dev	Median	Lower Quartile	Upper Quartile	Quartile Range	Minimum	Maximum
SMA type 2	L1	13	7.161	2.192	6.690	5.810	8.520	2.710	3.920	11.730
	L2	11	7.266	2.221	7.220	4.800	9.570	4.770	4.090	10.850
	L3	12	6.471	1.845	6.485	4.870	7.835	2.965	3.990	10.080
	L4	12	7.867	2.937	6.780	5.735	9.975	4.240	4.970	14.610
	M1	11	7.935	2.264	7.550	6.400	10.290	3.890	4.000	11.030
	M2	12	7.139	2.498	6.155	5.115	9.405	4.290	4.630	11.950
	M3	10	7.392	2.324	8.135	4.740	9.180	4.440	4.080	11.030
	M4	8	7.321	3.151	6.385	5.060	10.005	4.945	3.260	12.410
	M5	6	7.202	1.645	7.585	6.330	7.810	1.480	4.510	9.390
	M6	2	9.710	2.843	9.710	7.700	11.720	4.020	7.700	11.720
	M7	0	.	.	.	.	.	.	.	.
	M8	0	.	.	.	.	.	.	.	.
SMA type 3	L1	35	7.099	1.823	6.710	5.750	7.600	1.850	4.640	11.870
	L2	22	6.691	1.811	6.475	5.250	7.870	2.620	4.200	10.800
	L3	21	7.222	2.052	6.520	5.830	8.190	2.360	5.360	12.980
	L4	21	6.738	1.903	6.190	5.560	6.670	1.110	4.740	12.290
	M1	28	7.030	1.657	6.490	5.790	8.275	2.485	5.050	10.850
	M2	27	7.321	1.833	7.040	5.470	8.870	3.400	4.870	11.310
	M3	19	6.738	1.389	6.160	5.860	7.600	1.740	5.480	10.620
	M4	16	7.504	2.094	7.165	6.005	8.630	2.625	4.330	11.480
	M5	17	7.442	2.053	7.470	5.760	8.760	3.000	3.910	11.270
	M6	14	7.513	2.296	7.360	5.570	9.230	3.660	4.950	13.200
	M7	8	7.471	2.362	6.695	6.365	8.040	1.675	4.830	12.740
	M8	2	7.180	0.226	7.180	7.020	7.340	0.320	7.020	7.340
Total	L1	48	7.116	1.906	6.700	5.755	7.935	2.180	3.920	11.870
	L2	33	6.883	1.941	6.890	5.250	8.020	2.770	4.090	10.850
	L3	33	6.949	1.984	6.520	5.770	7.930	2.160	3.990	12.980
	L4	33	7.148	2.352	6.290	5.560	7.530	1.970	4.740	14.610
	M1	39	7.285	1.863	6.760	5.920	8.550	2.630	4.000	11.030
	M2	39	7.265	2.028	6.990	5.370	9.050	3.680	4.630	11.950
	M3	29	6.963	1.754	6.240	5.860	8.110	2.250	4.080	11.030
	M4	24	7.443	2.427	6.970	5.530	9.205	3.675	3.260	12.410
	M5	23	7.380	1.922	7.470	5.760	8.760	3.000	3.910	11.270
	M6	16	7.788	2.382	7.790	5.710	9.300	3.590	4.950	13.200
	M7	8	7.471	2.362	6.695	6.365	8.040	1.675	4.830	12.740
	M8	2	7.180	0.226	7.180	7.020	7.340	0.320	7.020	7.340

Table S1f. Descriptive statistics: Platelet count [number/nL].

	Injection	N	Mean	Std Dev	Median	Lower Quartile	Upper Quartile	Quartile Range	Minimum	Maximum
SMA type 2	L1	13	315.154	121.951	331.000	232.000	393.000	161.000	103.000	477.000
	L2	11	309.909	118.419	320.000	233.000	413.000	180.000	81.000	465.000
	L3	12	290.750	93.937	317.000	263.000	345.500	82.500	66.000	417.000
	L4	12	312.250	141.532	316.500	234.000	378.000	144.000	87.000	596.000
	M1	11	306.636	96.266	311.000	246.000	356.000	110.000	164.000	509.000
	M2	12	291.000	115.548	294.000	236.500	356.500	120.000	61.000	497.000
	M3	10	310.700	103.144	321.500	263.000	367.000	104.000	82.000	442.000
	M4	8	287.000	114.252	270.000	234.500	345.500	111.000	101.000	495.000
	M5	6	271.333	92.294	274.000	242.000	338.000	96.000	116.000	384.000
	M6	2	294.500	61.518	294.500	251.000	338.000	87.000	251.000	338.000
	M7	0	.	.	.	.	.	.	.	.
	M8	0	.	.	.	.	.	.	.	.
SMA type 3	L1	35	265.943	50.887	254.000	228.000	307.000	79.000	176.000	368.000
	L2	22	259.182	48.040	250.500	225.000	300.000	75.000	165.000	336.000
	L3	21	261.048	52.298	252.000	232.000	279.000	47.000	173.000	376.000
	L4	21	264.095	55.314	251.000	215.000	318.000	103.000	169.000	353.000
	M1	28	278.250	60.863	265.500	229.500	316.500	87.000	198.000	430.000
	M2	27	277.444	66.449	273.000	226.000	302.000	76.000	200.000	532.000
	M3	19	262.211	57.018	251.000	226.000	313.000	87.000	176.000	378.000
	M4	16	264.063	44.895	258.500	222.000	311.000	89.000	201.000	328.000
	M5	17	260.235	49.127	257.000	228.000	288.000	60.000	151.000	342.000
	M6	14	267.000	46.347	256.000	240.000	302.000	62.000	193.000	368.000
	M7	8	267.250	43.863	264.500	228.500	290.500	62.000	220.000	351.000
	M8	2	237.500	19.092	237.500	224.000	251.000	27.000	224.000	251.000
Total	L1	48	279.271	78.478	268.500	228.000	330.500	102.500	103.000	477.000
	L2	33	276.091	80.539	260.000	226.000	322.000	96.000	81.000	465.000
	L3	33	271.848	70.379	253.000	246.000	327.000	81.000	66.000	417.000
	L4	33	281.606	96.703	284.000	215.000	325.000	110.000	87.000	596.000
	M1	39	286.256	72.375	275.000	234.000	330.000	96.000	164.000	509.000
	M2	39	281.615	83.223	275.000	226.000	316.000	90.000	61.000	532.000
	M3	29	278.931	77.844	263.000	231.000	332.000	101.000	82.000	442.000
	M4	24	271.708	73.548	261.500	225.500	317.500	92.000	101.000	495.000
	M5	23	263.130	60.959	259.000	228.000	296.000	68.000	116.000	384.000
	M6	16	270.438	46.927	256.000	241.000	309.500	68.500	193.000	368.000
	M7	8	267.250	43.863	264.500	228.500	290.500	62.000	220.000	351.000
	M8	2	237.500	19.092	237.500	224.000	251.000	27.000	224.000	251.000

Table S1g. Descriptive statistics: International normalized ratio (INR).

SMA type	Injection	N	Mean	Std Dev	Median	Lower Quartile	Upper Quartile	Quartile Range	Minimum	Maximum
SMA type 2	L1	12	1.020	0.062	1.020	0.970	1.075	0.105	0.920	1.120
	L2	11	1.019	0.049	1.020	0.960	1.070	0.110	0.950	1.090
	L3	10	1.034	0.039	1.035	1.000	1.050	0.050	0.990	1.120
	L4	12	1.031	0.056	1.035	0.985	1.075	0.090	0.940	1.120
	M1	12	1.045	0.050	1.040	1.015	1.080	0.065	0.940	1.130
	M2	13	1.035	0.045	1.030	1.010	1.060	0.050	0.960	1.100
	M3	10	1.033	0.058	1.040	0.960	1.090	0.130	0.960	1.100
	M4	8	1.013	0.058	1.030	0.965	1.055	0.090	0.920	1.080
	M5	6	1.008	0.061	1.000	0.960	1.040	0.080	0.940	1.110
	M6	2	1.015	0.106	1.015	0.940	1.090	0.150	0.940	1.090
	M7	0	.	.	.	.	.	.	.	.
SMA type 3	L1	16	1.024	0.077	1.005	0.960	1.090	0.130	0.930	1.190
	L2	12	1.024	0.074	1.010	0.995	1.065	0.070	0.890	1.170
	L3	11	0.997	0.068	1.000	0.950	1.040	0.090	0.890	1.130
	L4	11	1.003	0.052	0.980	0.970	1.040	0.070	0.940	1.090
	M1	11	1.009	0.067	1.010	0.960	1.060	0.100	0.890	1.120
	M2	9	1.031	0.072	1.020	0.980	1.060	0.080	0.960	1.180
	M3	3	1.027	0.090	0.980	0.970	1.130	0.160	0.970	1.130
	M4	3	1.027	0.051	1.040	0.970	1.070	0.100	0.970	1.070
	M5	3	1.027	0.100	1.020	0.930	1.130	0.200	0.930	1.130
	M6	3	1.053	0.112	1.010	0.970	1.180	0.210	0.970	1.180
	M7	1	0.990	.	0.990	0.990	0.990	0.000	0.990	0.990
Total	L1	28	1.022	0.070	1.020	0.960	1.075	0.115	0.920	1.190
	L2	23	1.022	0.062	1.010	0.990	1.070	0.080	0.890	1.170
	L3	21	1.015	0.058	1.020	0.990	1.040	0.050	0.890	1.130
	L4	23	1.017	0.055	1.010	0.980	1.060	0.080	0.940	1.120
	M1	23	1.028	0.060	1.030	0.990	1.070	0.080	0.890	1.130
	M2	22	1.034	0.056	1.025	0.990	1.060	0.070	0.960	1.180
	M3	13	1.032	0.062	1.020	0.970	1.090	0.120	0.960	1.130
	M4	11	1.016	0.054	1.030	0.970	1.070	0.100	0.920	1.080
	M5	9	1.014	0.070	1.000	0.960	1.040	0.080	0.930	1.130
	M6	5	1.038	0.097	1.010	0.970	1.090	0.120	0.940	1.180
	M7	1	0.990	.	0.990	0.990	0.990	0.000	0.990	0.990

Table S1h. Descriptive statistics: Activated partial thromboplastin time (aPTT) [s].

SMA type	Injection	N	Mean	Std Dev	Median	Lower Quartile	Upper Quartile	Quartile Range	Minimum	Maximum
SMA type 2	L1	12	28.842	2.580	29.000	27.550	30.900	3.350	24.300	32.100
	L2	11	28.745	2.745	28.200	26.600	31.800	5.200	25.100	33.200
	L3	10	28.490	2.801	28.850	26.700	31.100	4.400	23.300	32.200
	L4	12	28.183	2.609	29.050	25.600	30.150	4.550	24.400	32.500
	M1	12	28.325	1.483	28.450	27.250	29.450	2.200	26.000	30.800
	M2	13	29.115	1.875	29.500	27.100	29.900	2.800	26.800	33.300
	M3	10	28.400	2.251	27.900	26.500	30.100	3.600	25.800	32.100
	M4	8	27.150	1.918	27.900	25.150	28.750	3.600	24.500	29.100
	M5	6	26.833	3.065	28.050	23.200	28.800	5.600	22.800	30.100
	M6	2	22.800	1.131	22.800	22.000	23.600	1.600	22.000	23.600
	M7	0	.	.	.	.	.	.	.	.
SMA type 3	L1	16	27.519	3.125	27.450	25.200	29.650	4.450	20.900	32.900
	L2	11	28.082	2.635	28.100	26.200	30.500	4.300	24.000	32.500
	L3	11	27.064	2.480	26.700	25.500	29.100	3.600	23.100	31.800
	L4	11	27.500	2.986	27.300	24.800	30.500	5.700	22.700	31.800
	M1	11	27.255	2.977	28.000	25.200	29.800	4.600	20.900	30.700
	M2	9	29.444	3.472	29.700	28.500	32.400	3.900	22.000	33.300
	M3	3	30.533	5.193	31.300	25.000	35.300	10.300	25.000	35.300
	M4	3	29.400	3.315	30.900	25.600	31.700	6.100	25.600	31.700
	M5	3	28.367	4.600	30.400	23.100	31.600	8.500	23.100	31.600
	M6	3	31.433	1.626	31.100	30.000	33.200	3.200	30.000	33.200
	M7	1	30.800	.	30.800	30.800	30.800	0.000	30.800	30.800
Total	L1	28	28.086	2.930	28.250	25.600	30.550	4.950	20.900	32.900
	L2	22	28.414	2.648	28.150	26.600	30.500	3.900	24.000	33.200
	L3	21	27.743	2.672	27.900	25.800	29.300	3.500	23.100	32.200
	L4	23	27.857	2.753	27.600	25.000	30.200	5.200	22.700	32.500
	M1	23	27.813	2.330	28.100	26.200	29.600	3.400	20.900	30.800
	M2	22	29.250	2.575	29.600	27.100	30.500	3.400	22.000	33.300
	M3	13	28.892	3.028	29.000	26.500	31.000	4.500	25.000	35.300
	M4	11	27.764	2.424	28.200	25.500	29.100	3.600	24.500	31.700
	M5	9	27.344	3.428	28.100	23.200	30.100	6.900	22.800	31.600
	M6	5	27.980	4.899	30.000	23.600	31.100	7.500	22.000	33.200
	M7	1	30.800	.	30.800	30.800	30.800	0.000	30.800	30.800

Table S1i. Descriptive statistics: Creatinine [mg/dL].

SMA type	Injection	N	Mean	Std Dev	Median	Lower Quartile	Upper Quartile	Quartile Range	Minimum	Maximum
SMA type 2	L1	13	0.308	0.048	0.300	0.270	0.320	0.050	0.260	0.430
	L2	11	0.288	0.050	0.280	0.260	0.320	0.060	0.190	0.370
	L3	12	0.303	0.047	0.290	0.270	0.330	0.060	0.250	0.410
	L4	11	0.306	0.051	0.300	0.270	0.340	0.070	0.250	0.420
	M1	11	0.306	0.061	0.310	0.270	0.340	0.070	0.190	0.410
	M2	11	0.351	0.120	0.320	0.280	0.350	0.070	0.230	0.620
	M3	10	0.292	0.074	0.295	0.280	0.310	0.030	0.160	0.450
	M4	9	0.294	0.097	0.320	0.220	0.370	0.150	0.150	0.430
	M5	6	0.378	0.134	0.385	0.280	0.480	0.200	0.210	0.530
	M6	2	0.245	0.134	0.245	0.150	0.340	0.190	0.150	0.340
	M7	0	.	.	.	.	.	.	.	.
	M8	0	.	.	.	.	.	.	.	.
SMA type 3	L1	33	0.492	0.141	0.510	0.350	0.630	0.280	0.280	0.780
	L2	22	0.503	0.168	0.525	0.350	0.660	0.310	0.270	0.810
	L3	20	0.438	0.168	0.390	0.310	0.510	0.200	0.230	0.890
	L4	21	0.456	0.147	0.390	0.340	0.550	0.210	0.250	0.740
	M1	27	0.437	0.144	0.380	0.320	0.580	0.260	0.180	0.660
	M2	27	0.457	0.151	0.400	0.310	0.600	0.290	0.290	0.760
	M3	19	0.483	0.153	0.500	0.350	0.600	0.250	0.200	0.730
	M4	16	0.519	0.154	0.535	0.350	0.665	0.315	0.290	0.700
	M5	17	0.498	0.161	0.520	0.330	0.640	0.310	0.200	0.720
	M6	14	0.441	0.189	0.430	0.320	0.520	0.200	0.150	0.810
	M7	8	0.449	0.163	0.510	0.325	0.540	0.215	0.180	0.660
	M8	2	0.460	0.226	0.460	0.300	0.620	0.320	0.300	0.620
Total	L1	46	0.440	0.148	0.390	0.310	0.540	0.230	0.260	0.780
	L2	33	0.432	0.173	0.360	0.300	0.550	0.250	0.190	0.810
	L3	32	0.387	0.150	0.340	0.280	0.455	0.175	0.230	0.890
	L4	32	0.404	0.141	0.340	0.300	0.535	0.235	0.250	0.740
	M1	38	0.399	0.139	0.340	0.310	0.530	0.220	0.180	0.660
	M2	38	0.426	0.149	0.345	0.310	0.560	0.250	0.230	0.760
	M3	29	0.417	0.159	0.350	0.300	0.550	0.250	0.160	0.730
	M4	25	0.438	0.173	0.390	0.320	0.610	0.290	0.150	0.700
	M5	23	0.467	0.160	0.480	0.320	0.610	0.290	0.200	0.720
	M6	16	0.416	0.191	0.405	0.295	0.515	0.220	0.150	0.810
	M7	8	0.449	0.163	0.510	0.325	0.540	0.215	0.180	0.660
	M8	2	0.460	0.226	0.460	0.300	0.620	0.320	0.300	0.620

Table S1j. Descriptive statistics: Urea nitrogen [mg/dL].

SMA type	Injection	N	Mean	Std Dev	Median	Lower Quartile	Upper Quartile	Quartile Range	Minimum	Maximum
SMA type 2	L1	13	8.769	4.126	8.000	6.000	10.000	4.000	4.000	18.000
	L2	11	9.000	3.661	8.000	6.000	13.000	7.000	4.000	15.000
	L3	12	9.417	3.423	8.500	7.000	11.500	4.500	5.000	16.000
	L4	11	10.273	5.424	8.000	7.000	12.000	5.000	5.000	23.000
	M1	11	10.609	3.427	11.000	9.000	13.000	4.000	3.000	15.700
	M2	11	11.427	4.928	11.000	7.000	16.000	9.000	6.000	21.000
	M3	10	9.170	3.627	8.500	7.000	11.000	4.000	4.000	16.000
	M4	9	8.733	3.297	7.000	6.400	11.000	4.600	5.000	15.000
	M5	6	10.750	5.383	10.000	8.500	10.000	1.500	5.000	21.000
	M6	2	9.550	0.495	9.550	9.200	9.900	0.700	9.200	9.900
	M7	0	.	.	.	.	.	.	.	.
	M8	0	.	.	.	.	.	.	.	.
SMA type 3	L1	33	11.909	4.318	12.000	9.000	14.000	5.000	5.000	22.000
	L2	22	12.295	4.672	11.500	9.000	13.000	4.000	7.000	26.000
	L3	20	11.625	3.814	10.500	9.000	13.500	4.500	6.000	21.000
	L4	21	11.571	3.804	12.000	9.000	14.000	5.000	5.000	20.000
	M1	26	11.112	4.033	10.000	8.000	13.000	5.000	5.000	21.900
	M2	27	12.222	3.639	11.000	10.000	15.300	5.300	6.000	20.000
	M3	19	12.232	3.012	13.000	9.000	15.100	6.100	8.000	16.000
	M4	16	11.250	2.595	11.000	9.500	12.500	3.000	7.000	17.000
	M5	17	12.059	2.580	13.000	11.000	13.600	2.600	5.000	15.000
	M6	14	11.843	3.345	12.950	9.700	14.000	4.300	6.400	18.000
	M7	8	13.013	3.549	14.100	9.700	14.550	4.850	8.400	19.000
	M8	2	10.100	0.566	10.100	9.700	10.500	0.800	9.700	10.500
Total	L1	46	11.022	4.455	10.000	8.000	14.000	6.000	4.000	22.000
	L2	33	11.197	4.582	11.000	8.000	13.000	5.000	4.000	26.000
	L3	32	10.797	3.776	10.000	8.000	13.000	5.000	5.000	21.000
	L4	32	11.125	4.384	11.500	8.000	13.800	5.800	5.000	23.000
	M1	37	10.962	3.822	10.000	9.000	13.000	4.000	3.000	21.900
	M2	38	11.992	4.000	11.000	9.300	15.300	6.000	6.000	21.000
	M3	29	11.176	3.500	11.000	9.000	14.000	5.000	4.000	16.000
	M4	25	10.344	3.058	11.000	8.000	12.000	4.000	5.000	17.000
	M5	23	11.717	3.431	12.000	10.000	13.600	3.600	5.000	21.000
	M6	16	11.556	3.214	11.800	9.450	14.000	4.550	6.400	18.000
	M7	8	13.013	3.549	14.100	9.700	14.550	4.850	8.400	19.000
	M8	2	10.100	0.566	10.100	9.700	10.500	0.800	9.700	10.500

Table S1k. Descriptive statistics: Aspartate aminotransferase (AST) [U/L].

SMA type	Injection	N	Mean	Std Dev	Median	Lower Quartile	Upper Quartile	Quartile Range	Minimum	Maximum
SMA type 2	L1	13	21.000	8.926	19.000	15.000	26.000	11.000	11.000	39.000
	L2	11	16.727	6.930	15.000	11.000	24.000	13.000	9.000	30.000
	L3	12	18.500	9.318	15.500	12.000	23.500	11.500	8.000	41.000
	L4	11	18.182	7.653	17.000	14.000	22.000	8.000	9.000	38.000
	M1	11	16.091	8.227	14.000	12.000	16.000	4.000	6.000	36.000
	M2	10	18.500	8.236	17.500	12.000	23.000	11.000	7.000	34.000
	M3	10	17.900	10.170	14.000	10.000	24.000	14.000	7.000	38.000
	M4	9	17.667	6.103	17.000	13.000	20.000	7.000	12.000	30.000
	M5	6	17.500	7.007	15.500	13.000	18.000	5.000	12.000	31.000
	M6	2	29.500	6.364	29.500	25.000	34.000	9.000	25.000	34.000
	M7	0	.	.	.	.	.	.	.	.
	M8	0	.	.	.	.	.	.	.	.
SMA type 3	L1	33	28.485	13.067	25.000	18.000	36.000	18.000	11.000	72.000
	L2	22	23.409	10.326	22.000	16.000	31.000	15.000	7.000	43.000
	L3	20	23.650	11.962	20.500	14.000	29.000	15.000	10.000	52.000
	L4	21	26.000	11.798	22.000	17.000	35.000	18.000	13.000	59.000
	M1	26	24.577	8.686	23.000	17.000	30.000	13.000	14.000	49.000
	M2	27	28.815	24.595	23.000	19.000	28.000	9.000	12.000	142.000
	M3	19	30.684	24.752	23.000	19.000	33.000	14.000	14.000	124.000
	M4	16	32.688	19.969	28.000	20.500	39.500	19.000	12.000	92.000
	M5	17	35.118	25.522	28.000	19.000	39.000	20.000	9.000	111.000
	M6	14	31.500	19.417	26.500	18.000	35.000	17.000	15.000	88.000
	M7	8	38.000	25.428	28.500	20.500	49.000	28.500	16.000	92.000
	M8	2	32.500	12.021	32.500	24.000	41.000	17.000	24.000	41.000
Total	L1	46	26.370	12.421	24.500	17.000	33.000	16.000	11.000	72.000
	L2	33	21.182	9.758	20.000	13.000	27.000	14.000	7.000	43.000
	L3	32	21.719	11.177	20.000	13.500	25.500	12.000	8.000	52.000
	L4	32	23.313	11.087	19.000	16.000	28.500	12.500	9.000	59.000
	M1	37	22.054	9.309	20.000	16.000	29.000	13.000	6.000	49.000
	M2	37	26.027	21.804	22.000	17.000	27.000	10.000	7.000	142.000
	M3	29	26.276	21.572	22.000	15.000	31.000	16.000	7.000	124.000
	M4	25	27.280	17.771	22.000	16.000	31.000	15.000	12.000	92.000
	M5	23	30.522	23.397	25.000	15.000	34.000	19.000	9.000	111.000
	M6	16	31.250	18.164	26.500	19.500	34.500	15.000	15.000	88.000
	M7	8	38.000	25.428	28.500	20.500	49.000	28.500	16.000	92.000
	M8	2	32.500	12.021	32.500	24.000	41.000	17.000	24.000	41.000

Table S1l. Descriptive statistics: Alanine aminotransferase (ALT) [U/L].

SMA type	Injection	N	Mean	Std Dev	Median	Lower Quartile	Upper Quartile	Quartile Range	Minimum	Maximum
SMA type 2	L1	13	31.538	29.105	17.000	16.000	34.000	18.000	8.000	111.000
	L2	11	28.636	27.292	17.000	13.000	39.000	26.000	8.000	99.000
	L3	12	25.917	24.956	14.500	12.500	31.000	18.500	10.000	94.000
	L4	11	26.182	24.082	17.000	10.000	28.000	18.000	8.000	90.000
	M1	11	24.909	28.483	12.000	10.000	38.000	28.000	9.000	103.000
	M2	10	30.600	25.954	19.000	12.000	40.000	28.000	10.000	90.000
	M3	10	30.100	30.824	15.000	11.000	34.000	23.000	10.000	101.000
	M4	9	26.444	23.222	17.000	12.000	40.000	28.000	6.000	76.000
	M5	6	27.333	13.292	26.000	17.000	37.000	20.000	13.000	45.000
	M6	2	35.500	2.121	35.500	34.000	37.000	3.000	34.000	37.000
	M7	0	.	.	.	.	.	.	.	.
	M8	0	.	.	.	.	.	.	.	.
SMA type 3	L1	33	47.727	24.240	38.000	32.000	64.000	32.000	17.000	118.000
	L2	22	39.727	20.972	31.000	25.000	57.000	32.000	14.000	98.000
	L3	20	45.800	27.604	35.000	25.000	58.500	33.500	17.000	119.000
	L4	21	40.333	19.430	34.000	28.000	47.000	19.000	21.000	86.000
	M1	26	38.038	18.010	34.000	28.000	46.000	18.000	11.000	88.000
	M2	27	47.296	46.959	33.000	27.000	47.000	20.000	16.000	265.000
	M3	19	49.158	47.318	36.000	27.000	49.000	22.000	15.000	228.000
	M4	16	55.438	37.331	44.000	31.500	71.000	39.500	18.000	162.000
	M5	17	56.588	42.726	41.000	33.000	80.000	47.000	14.000	193.000
	M6	14	51.857	29.838	47.500	30.000	58.000	28.000	17.000	125.000
	M7	8	63.875	51.271	47.000	33.000	79.000	46.000	15.000	178.000
	M8	2	48.000	21.213	48.000	33.000	63.000	30.000	33.000	63.000
Total	L1	46	43.152	26.421	35.000	23.000	60.000	37.000	8.000	118.000
	L2	33	36.030	23.443	28.000	18.000	53.000	35.000	8.000	99.000
	L3	32	38.344	27.994	29.500	17.500	50.000	32.500	10.000	119.000
	L4	32	35.469	21.847	28.000	23.000	44.500	21.500	8.000	90.000
	M1	37	34.135	22.082	32.000	18.000	43.000	25.000	9.000	103.000
	M2	37	42.784	42.632	30.000	23.000	47.000	24.000	10.000	265.000
	M3	29	42.586	42.776	28.000	19.000	48.000	29.000	10.000	228.000
	M4	25	45.000	35.391	38.000	18.000	52.000	34.000	6.000	162.000
	M5	23	48.957	39.247	37.000	31.000	48.000	17.000	13.000	193.000
	M6	16	49.813	28.339	45.000	32.000	56.500	24.500	17.000	125.000
	M7	8	63.875	51.271	47.000	33.000	79.000	46.000	15.000	178.000
	M8	2	48.000	21.213	48.000	33.000	63.000	30.000	33.000	63.000

Table S1m. Descriptive statistics: Gamma-glutamyltransferase (GGT) [U/L].

SMA type	Injection	N	Mean	Std Dev	Median	Lower Quartile	Upper Quartile	Quartile Range	Minimum	Maximum
SMA type 2	L1	13	36.077	45.106	20.000	15.000	30.000	15.000	7.000	176.000
	L2	11	33.091	39.162	17.000	11.000	34.000	23.000	7.000	142.000
	L3	12	34.167	48.867	18.000	11.000	27.500	16.500	7.000	181.000
	L4	11	28.091	23.067	23.000	11.000	39.000	28.000	7.000	73.000
	M1	11	29.636	36.319	15.000	13.000	27.000	14.000	7.000	126.000
	M2	10	46.000	53.398	21.000	18.000	64.000	46.000	10.000	185.000
	M3	10	43.300	62.945	16.000	11.000	58.000	47.000	9.000	214.000
	M4	9	43.667	54.231	22.000	13.000	50.000	37.000	9.000	174.000
	M5	6	31.667	34.604	22.000	12.000	25.000	13.000	8.000	101.000
	M6	2	25.000	2.828	25.000	23.000	27.000	4.000	23.000	27.000
	M7	0	.	.	.	.	.	.	.	.
	M8	0	.	.	.	.	.	.	.	.
SMA type 3	L1	33	43.697	39.329	32.000	20.000	50.000	30.000	7.000	190.000
	L2	22	35.091	30.611	27.000	17.000	39.000	22.000	12.000	145.000
	L3	20	42.700	45.299	28.000	17.500	46.000	28.500	11.000	216.000
	L4	21	32.619	22.074	27.000	18.000	40.000	22.000	9.000	93.000
	M1	26	35.154	37.404	25.000	16.000	39.000	23.000	10.000	190.000
	M2	27	39.296	32.214	26.000	16.000	53.000	37.000	11.000	130.000
	M3	19	35.579	30.204	20.000	16.000	45.000	29.000	7.000	111.000
	M4	16	48.563	43.273	29.000	19.000	71.000	52.000	12.000	143.000
	M5	17	48.235	41.050	31.000	20.000	53.000	33.000	10.000	148.000
	M6	14	46.786	44.430	33.000	19.000	50.000	31.000	11.000	167.000
	M7	8	59.625	45.396	34.500	26.500	106.000	79.500	16.000	127.000
	M8	2	49.500	37.477	49.500	23.000	76.000	53.000	23.000	76.000
Total	L1	46	41.543	40.676	27.500	16.000	50.000	34.000	7.000	190.000
	L2	33	34.424	33.093	26.000	14.000	35.000	21.000	7.000	145.000
	L3	32	39.500	46.072	25.000	15.500	44.000	28.500	7.000	216.000
	L4	32	31.063	22.153	23.500	16.500	39.500	23.000	7.000	93.000
	M1	37	33.514	36.668	20.000	15.000	32.000	17.000	7.000	190.000
	M2	37	41.108	38.359	25.000	18.000	53.000	35.000	10.000	185.000
	M3	29	38.241	43.289	20.000	13.000	45.000	32.000	7.000	214.000
	M4	25	46.800	46.437	26.000	16.000	50.000	34.000	9.000	174.000
	M5	23	43.913	39.408	25.000	19.000	53.000	34.000	8.000	148.000
	M6	16	44.063	42.032	29.500	20.000	44.500	24.500	11.000	167.000
	M7	8	59.625	45.396	34.500	26.500	106.000	79.500	16.000	127.000
	M8	2	49.500	37.477	49.500	23.000	76.000	53.000	23.000	76.000

Table S2a. Change from baseline (L1): Cerebrospinal fluid (CSF) - White blood cell (WBC) count [number/µL].

Injection	SMA type	N	Mean	Std Dev	Median	Lower Quartile	Upper Quartile	Quartile Range	Min	Max	p-value*
L2	SMA type 2	9	0.000	1.5000	0.000	-1.000	1.000	2.000	-2.000	3.000	1.0000
L2	SMA type 3	33	0.394	3.2972	1.000	0.000	2.000	2.000	-12.000	9.000	0.1078
L2	Total	42	0.310	2.9918	0.000	-1.000	2.000	3.000	-12.000	9.000	0.2153
L3	SMA type 2	12	1.667	4.4992	0.500	-1.000	2.000	3.000	-4.000	12.000	0.7539
L3	SMA type 3	30	0.667	1.6884	1.000	0.000	2.000	2.000	-4.000	4.000	0.0525
L3	Total	42	0.952	2.7670	1.000	0.000	2.000	2.000	-4.000	12.000	0.0501
L4	SMA type 2	10	-0.400	2.1187	-0.500	-1.000	1.000	2.000	-5.000	2.000	1.0000
L4	SMA type 3	33	-0.182	1.6096	0.000	-1.000	1.000	2.000	-4.000	4.000	0.8238
L4	Total	43	-0.233	1.7160	0.000	-1.000	1.000	2.000	-5.000	4.000	0.7111
M1	SMA type 2	10	-0.100	1.2867	0.000	-1.000	0.000	1.000	-2.000	3.000	0.6250
M1	SMA type 3	29	-0.069	1.8113	0.000	-1.000	1.000	2.000	-4.000	4.000	0.8145
M1	Total	39	-0.077	1.6762	0.000	-1.000	0.000	1.000	-4.000	4.000	0.5235
M2	SMA type 2	8	-2.000	4.5356	-0.500	-1.500	0.000	1.500	-13.000	1.000	0.3750
M2	SMA type 3	24	-0.792	2.7502	0.000	-2.000	1.000	3.000	-11.000	4.000	0.8036
M2	Total	32	-1.094	3.2464	0.000	-2.000	0.500	2.500	-13.000	4.000	0.3833
M3	SMA type 2	12	-0.333	1.3707	0.000	-1.500	1.000	2.500	-3.000	1.000	1.0000
M3	SMA type 3	17	0.176	5.1990	0.000	-1.000	1.000	2.000	-12.000	16.000	1.0000
M3	Total	29	-0.034	4.0310	0.000	-1.000	1.000	2.000	-12.000	16.000	1.0000
M4	SMA type 2	8	-0.375	1.5059	-0.500	-1.000	0.500	1.500	-3.000	2.000	0.6875
M4	SMA type 3	16	-0.563	3.4635	0.000	-0.500	1.000	1.500	-12.000	3.000	0.7539
M4	Total	24	-0.500	2.9192	0.000	-1.000	1.000	2.000	-12.000	3.000	1.0000
M5	SMA type 2	7	0.286	1.7995	1.000	-1.000	2.000	3.000	-3.000	2.000	0.6875
M5	SMA type 3	15	-1.333	3.2878	0.000	-3.000	0.000	3.000	-11.000	4.000	0.1797
M5	Total	22	-0.818	2.9542	0.000	-2.000	1.000	3.000	-11.000	4.000	0.6072
M6	SMA type 2	3	0.000	1.0000	0.000	-1.000	1.000	2.000	-1.000	1.000	1.0000
M6	SMA type 3	13	-1.308	3.6603	-1.000	-2.000	0.000	2.000	-11.000	5.000	0.3438
M6	Total	16	-1.063	3.3360	-0.500	-2.000	0.500	2.500	-11.000	5.000	0.3877
M7	SMA type 2	1	-3.000	.	-3.000	-3.000	-3.000	0.000	-3.000	-3.000	1.0000
M7	SMA type 3	8	-1.625	2.9731	-1.500	-2.000	0.000	2.000	-8.000	2.000	0.2891
M7	Total	9	-1.778	2.8186	-2.000	-2.000	-1.000	1.000	-8.000	2.000	0.1797
M8	SMA type 3	2	-1.500	2.1213	-1.500	-3.000	0.000	3.000	-3.000	0.000	1.0000
M8	Total	2	-1.500	2.1213	-1.500	-3.000	0.000	3.000	-3.000	0.000	1.0000
Last available	SMA type 2	12	0.167	1.7495	0.500	-0.500	1.500	2.000	-3.000	2.000	0.5078
Last available	SMA type 3	34	-0.382	2.2964	0.000	-2.000	0.000	2.000	-8.000	5.000	0.5034
Last available	Total	46	-0.239	2.1621	0.000	-1.000	1.000	2.000	-8.000	5.000	1.0000
***p-value of sign test**											

Table S2b. Change from baseline (L1): Cerebrospinal fluid (CSF) - Glucose [mg/dL].

Injection	SMA type	N	Mean	Std Dev	Median	Lower Quartile	Upper Quartile	Quartile Range	Min	Max	p-value*
L2	SMA type 2	9	-3.000	5.3385	-1.000	-6.000	1.000	7.000	-13.000	3.000	0.7266
L2	SMA type 3	33	-0.788	4.2994	-1.000	-4.000	2.000	6.000	-12.000	7.000	0.4869
L2	Total	42	-1.262	4.5642	-1.000	-4.000	2.000	6.000	-13.000	7.000	0.3489
L3	SMA type 2	12	-0.500	3.4245	-1.000	-2.500	1.500	4.000	-5.000	7.000	0.5488
L3	SMA type 3	29	-0.897	3.1774	-1.000	-3.000	1.000	4.000	-6.000	5.000	0.1686
L3	Total	41	-0.780	3.2133	-1.000	-3.000	1.000	4.000	-6.000	7.000	0.0989
L4	SMA type 2	10	-0.600	5.1683	-0.500	-3.000	4.000	7.000	-10.000	6.000	1.0000
L4	SMA type 3	33	-1.030	3.8119	-1.000	-2.000	1.000	3.000	-12.000	6.000	0.5847
L4	Total	43	-0.930	4.1022	-1.000	-3.000	1.000	4.000	-12.000	6.000	0.5224
M1	SMA type 2	10	-3.100	4.5326	-3.500	-5.000	0.000	5.000	-10.000	4.000	0.1797
M1	SMA type 3	29	-2.276	5.6940	-2.000	-5.000	1.000	6.000	-19.000	10.000	0.0522
M1	Total	39	-2.487	5.3748	-2.000	-5.000	1.000	6.000	-19.000	10.000	0.0113
M2	SMA type 2	9	-2.111	4.4001	-2.000	-5.000	1.000	6.000	-9.000	5.000	0.7266
M2	SMA type 3	23	-3.174	3.4857	-3.000	-5.000	-1.000	4.000	-10.000	3.000	0.0015
M2	Total	32	-2.875	3.7222	-3.000	-5.000	0.000	5.000	-10.000	5.000	0.0023
M3	SMA type 2	12	-2.500	5.3852	-2.000	-5.000	0.500	5.500	-13.000	6.000	0.2266
M3	SMA type 3	17	-2.353	4.8598	-2.000	-6.000	1.000	7.000	-11.000	7.000	0.2101
M3	Total	29	-2.414	4.9894	-2.000	-6.000	1.000	7.000	-13.000	7.000	0.0522
M4	SMA type 2	8	-3.750	6.1586	-2.000	-7.500	-0.500	7.000	-15.000	5.000	0.1250
M4	SMA type 3	16	-3.125	3.7572	-3.000	-6.500	-0.500	6.000	-10.000	5.000	0.0129
M4	Total	24	-3.333	4.5651	-3.000	-6.500	-0.500	6.000	-15.000	5.000	0.0015
M5	SMA type 2	7	-6.000	8.4853	-7.000	-13.000	-2.000	11.000	-15.000	10.000	0.1250
M5	SMA type 3	15	-3.800	2.9809	-4.000	-6.000	-2.000	4.000	-10.000	2.000	0.0010
M5	Total	22	-4.500	5.2531	-4.000	-7.000	-2.000	5.000	-15.000	10.000	0.0001
M6	SMA type 2	3	-8.333	10.7858	-13.000	-16.000	4.000	20.000	-16.000	4.000	1.0000
M6	SMA type 3	13	-2.769	4.3618	-2.000	-6.000	0.000	6.000	-11.000	4.000	0.2266
M6	Total	16	-3.813	5.9802	-2.500	-7.500	0.500	8.000	-16.000	4.000	0.1796
M7	SMA type 2	1	-10.000	.	-10.000	-10.000	-10.000	0.000	-10.000	-10.000	1.0000
M7	SMA type 3	8	-2.750	6.2507	-4.500	-6.500	1.000	7.500	-11.000	9.000	0.7266
M7	Total	9	-3.556	6.3268	-6.000	-7.000	1.000	8.000	-11.000	9.000	0.5078
M8	SMA type 3	2	-2.000	2.8284	-2.000	-4.000	0.000	4.000	-4.000	0.000	1.0000
M8	Total	2	-2.000	2.8284	-2.000	-4.000	0.000	4.000	-4.000	0.000	1.0000
Last available	SMA type 2	12	-1.667	7.5839	-0.500	-7.000	4.000	11.000	-16.000	10.000	0.7539
Last available	SMA type 3	34	-2.088	5.7807	-2.000	-6.000	1.000	7.000	-19.000	10.000	0.0428
Last available	Total	46	-1.978	6.2129	-1.500	-6.000	1.000	7.000	-19.000	10.000	0.0385

***p-value of sign test**

Table S2c. Change from baseline (L1): Cerebrospinal fluid (CSF) - Lactate [mmol/L].

Injection	SMA type	N	Mean	Std Dev	Median	Lower Quartile	Upper Quartile	Quartile Range	Min	Max	p-value*
L2	SMA type 2	9	0.033	0.1658	0.000	0.000	0.100	0.100	-0.200	0.300	0.6875
L2	SMA type 3	33	-0.024	0.1838	0.000	-0.200	0.100	0.300	-0.400	0.400	0.7111
L2	Total	42	-0.012	0.1797	0.000	-0.200	0.100	0.300	-0.400	0.400	1.0000
L3	SMA type 2	12	0.092	0.1676	0.100	-0.100	0.250	0.350	-0.100	0.300	0.5488
L3	SMA type 3	29	0.034	0.2208	0.100	-0.200	0.200	0.400	-0.400	0.500	0.5572
L3	Total	41	0.051	0.2063	0.100	-0.100	0.200	0.300	-0.400	0.500	0.3240
L4	SMA type 2	10	0.070	0.1252	0.050	0.000	0.200	0.200	-0.100	0.200	0.4531
L4	SMA type 3	33	0.015	0.1970	0.100	-0.100	0.100	0.200	-0.400	0.400	0.5847
L4	Total	43	0.028	0.1830	0.100	-0.100	0.200	0.300	-0.400	0.400	0.3240
M1	SMA type 2	10	0.100	0.2000	0.100	-0.100	0.300	0.400	-0.200	0.400	0.5078
M1	SMA type 3	29	0.062	0.2933	0.000	-0.100	0.200	0.300	-0.800	0.800	0.6476
M1	Total	39	0.072	0.2704	0.000	-0.100	0.200	0.300	-0.800	0.800	0.3449
M2	SMA type 2	9	-0.022	0.1302	0.000	-0.100	0.100	0.200	-0.300	0.100	1.0000
M2	SMA type 3	23	-0.039	0.1994	0.000	-0.200	0.100	0.300	-0.500	0.300	1.0000
M2	Total	32	-0.034	0.1807	0.000	-0.200	0.100	0.300	-0.500	0.300	1.0000
M3	SMA type 2	12	0.092	0.2151	0.100	0.000	0.250	0.250	-0.400	0.400	0.1797
M3	SMA type 3	17	0.041	0.2123	0.100	0.000	0.200	0.200	-0.400	0.400	0.1796
M3	Total	29	0.062	0.2111	0.100	0.000	0.200	0.200	-0.400	0.400	0.0347
M4	SMA type 2	8	0.038	0.2134	0.100	-0.050	0.150	0.200	-0.400	0.300	0.4531
M4	SMA type 3	16	0.100	0.2280	0.100	0.000	0.200	0.200	-0.400	0.600	0.1460
M4	Total	24	0.079	0.2206	0.100	0.000	0.200	0.200	-0.400	0.600	0.0636
M5	SMA type 2	7	0.029	0.1976	0.000	-0.100	0.200	0.300	-0.300	0.300	1.0000
M5	SMA type 3	15	0.093	0.2017	0.100	-0.100	0.200	0.300	-0.200	0.500	0.1796
M5	Total	22	0.073	0.1980	0.100	-0.100	0.200	0.300	-0.300	0.500	0.1671
M6	SMA type 2	3	-0.067	0.2082	0.000	-0.300	0.100	0.400	-0.300	0.100	1.0000
M6	SMA type 3	13	-0.031	0.1843	0.000	-0.200	0.100	0.300	-0.300	0.400	0.7539
M6	Total	16	-0.038	0.1821	0.000	-0.200	0.100	0.300	-0.300	0.400	0.7744
M7	SMA type 2	1	0.000	.	0.000	0.000	0.000	0.000	0.000	0.000	
M7	SMA type 3	8	-0.050	0.1852	-0.100	-0.150	0.050	0.200	-0.300	0.300	0.4531
M7	Total	9	-0.044	0.1740	-0.100	-0.100	0.000	0.100	-0.300	0.300	0.4531
M8	SMA type 3	2	-0.150	0.3536	-0.150	-0.400	0.100	0.500	-0.400	0.100	1.0000
M8	Total	2	-0.150	0.3536	-0.150	-0.400	0.100	0.500	-0.400	0.100	1.0000
Last available	SMA type 2	12	0.100	0.1859	0.150	0.000	0.250	0.250	-0.300	0.300	0.1797
Last available	SMA type 3	34	0.006	0.2103	0.000	-0.100	0.100	0.200	-0.500	0.400	0.6900
Last available	Total	46	0.030	0.2064	0.000	-0.100	0.200	0.300	-0.500	0.400	0.2295

***p-value of sign test**

Table S2d. Change from baseline (L1): Cerebrospinal fluid (CSF) – Total protein [mg/dL].

Injection	SMA type	N	Mean	Std Dev	Median	Lower Quartile	Upper Quartile	Quartile Range	Min	Max	p-value*
L2	SMA type 2	9	2.556	3.1667	1.000	0.000	6.000	6.000	-1.000	7.000	0.2188
L2	SMA type 3	33	0.848	5.3626	1.000	-1.000	4.000	5.000	-14.000	9.000	0.2649
L2	Total	42	1.214	4.9904	1.000	-1.000	5.000	6.000	-14.000	9.000	0.0895
L3	SMA type 2	12	1.750	4.4339	2.000	0.000	5.500	5.500	-8.000	7.000	0.1094
L3	SMA type 3	29	0.138	5.3031	1.000	0.000	2.000	2.000	-16.000	12.000	0.0784
L3	Total	41	0.610	5.0640	1.000	0.000	3.000	3.000	-16.000	12.000	0.0107
L4	SMA type 2	10	-1.500	4.0893	-0.500	-2.000	1.000	3.000	-11.000	2.000	1.0000
L4	SMA type 3	33	0.121	5.9569	0.000	-3.000	2.000	5.000	-11.000	22.000	0.7111
L4	Total	43	-0.256	5.5767	0.000	-3.000	2.000	5.000	-11.000	22.000	0.6271
M1	SMA type 2	10	2.600	7.8060	1.500	0.000	5.000	5.000	-10.000	18.000	0.1797
M1	SMA type 3	29	-0.138	5.3567	1.000	-2.000	4.000	6.000	-18.000	8.000	1.0000
M1	Total	39	0.564	6.0862	1.000	-2.000	4.000	6.000	-18.000	18.000	0.4177
M2	SMA type 2	9	7.111	11.6667	4.000	1.000	13.000	12.000	-7.000	32.000	0.1797
M2	SMA type 3	23	0.696	6.8120	1.000	-2.000	5.000	7.000	-17.000	13.000	0.5034
M2	Total	32	2.500	8.7547	2.000	-2.000	6.000	8.000	-17.000	32.000	0.1360
M3	SMA type 2	12	6.417	13.5878	3.500	1.000	11.500	10.500	-15.000	41.000	0.0654
M3	SMA type 3	17	1.353	4.1524	1.000	-1.000	5.000	6.000	-6.000	9.000	0.8036
M3	Total	29	3.448	9.4248	2.000	-1.000	6.000	7.000	-15.000	41.000	0.1221
M4	SMA type 2	8	6.500	11.6374	5.500	1.000	9.000	8.000	-10.000	31.000	0.0703
M4	SMA type 3	16	3.063	5.1182	2.500	0.500	5.500	5.000	-7.000	13.000	0.0352
M4	Total	24	4.208	7.8129	3.000	1.000	7.500	6.500	-10.000	31.000	0.0026
M5	SMA type 2	7	4.571	9.8125	2.000	-4.000	13.000	17.000	-5.000	22.000	1.0000
M5	SMA type 3	15	4.133	6.6961	5.000	-3.000	9.000	12.000	-7.000	18.000	0.1185
M5	Total	22	4.273	7.5793	5.000	-3.000	9.000	12.000	-7.000	22.000	0.1338
M6	SMA type 2	3	0.333	1.5275	0.000	-1.000	2.000	3.000	-1.000	2.000	1.0000
M6	SMA type 3	13	4.077	5.8660	2.000	0.000	6.000	6.000	-3.000	15.000	0.1460
M6	Total	16	3.375	5.4879	2.000	-0.500	6.000	6.500	-3.000	15.000	0.1796
M7	SMA type 2	1	-2.000	.	-2.000	-2.000	-2.000	0.000	-2.000	-2.000	1.0000
M7	SMA type 3	8	3.000	6.5247	5.000	1.000	7.000	6.000	-11.000	9.000	0.2891
M7	Total	9	2.444	6.3268	5.000	-2.000	7.000	9.000	-11.000	9.000	0.5078
M8	SMA type 3	2	7.000	7.0711	7.000	2.000	12.000	10.000	2.000	12.000	0.5000
M8	Total	2	7.000	7.0711	7.000	2.000	12.000	10.000	2.000	12.000	0.5000
Last available	SMA type 2	12	3.833	7.9639	1.000	-1.500	9.000	10.500	-5.000	22.000	0.7539
Last available	SMA type 3	34	2.588	5.4111	4.000	-1.000	6.000	7.000	-11.000	12.000	0.0135
Last available	Total	46	2.913	6.1058	3.000	-1.000	6.000	7.000	-11.000	22.000	0.0137
***p-value of sign test**											

Table S2e. Change from baseline (L1): White blood cell (WBC) count [number/nL].

Injection	SMA type	N	Mean	Std Dev	Median	Lower Quartile	Upper Quartile	Quartile Range	Min	Max	p-value*
L2	SMA type 2	11	0.223	1.3142	0.110	-0.180	0.540	0.720	-2.160	2.560	1.0000
L2	SMA type 3	21	-0.266	1.4522	-0.130	-1.100	0.610	1.710	-3.150	2.960	1.0000
L2	Total	32	-0.098	1.4047	-0.030	-1.015	0.575	1.590	-3.150	2.960	1.0000
L3	SMA type 2	11	-0.252	1.5183	0.090	-0.630	0.680	1.310	-3.740	1.930	1.0000
L3	SMA type 3	21	0.038	1.3307	-0.040	-0.820	0.870	1.690	-2.780	2.930	1.0000
L3	Total	32	-0.062	1.3805	0.025	-0.725	0.855	1.580	-3.740	2.930	1.0000
L4	SMA type 2	11	0.919	1.9901	0.900	-0.200	1.310	1.510	-1.200	6.090	1.0000
L4	SMA type 3	20	-0.252	1.2651	-0.530	-0.970	0.440	1.410	-2.680	3.160	0.2632
L4	Total	31	0.164	1.6303	-0.110	-0.800	1.050	1.850	-2.680	6.090	0.4731
M1	SMA type 2	10	0.701	1.0660	0.725	0.330	0.980	0.650	-1.440	2.430	0.1094
M1	SMA type 3	27	-0.007	0.9681	-0.060	-0.630	0.620	1.250	-1.660	2.890	0.7011
M1	Total	37	0.185	1.0307	0.080	-0.510	0.790	1.300	-1.660	2.890	0.7428
M2	SMA type 2	11	0.029	1.0646	0.220	-0.750	0.710	1.460	-2.010	1.780	0.5488
M2	SMA type 3	26	0.100	1.3079	0.200	-0.310	0.730	1.040	-3.100	2.850	0.5572
M2	Total	37	0.079	1.2263	0.220	-0.310	0.710	1.020	-3.100	2.850	0.3240
M3	SMA type 2	10	0.255	2.2978	0.015	-0.460	1.830	2.290	-3.710	4.340	1.0000
M3	SMA type 3	18	-0.536	1.9598	0.005	-1.230	0.540	1.770	-6.390	1.630	1.0000
M3	Total	28	-0.254	2.0802	0.005	-1.035	0.635	1.670	-6.390	4.340	1.0000
M4	SMA type 2	8	0.013	1.8450	-0.240	-1.205	0.575	1.780	-2.050	3.890	1.0000
M4	SMA type 3	15	0.413	1.4211	0.310	-0.150	1.060	1.210	-2.280	3.880	0.1185
M4	Total	23	0.273	1.5512	0.260	-0.780	0.970	1.750	-2.280	3.890	0.2100
M5	SMA type 2	6	-0.618	1.8003	-0.060	-2.340	0.850	3.190	-3.200	1.100	1.0000
M5	SMA type 3	16	0.067	1.2751	0.240	-0.630	1.055	1.685	-2.620	2.050	0.8036
M5	Total	22	-0.120	1.4250	0.240	-0.730	0.850	1.580	-3.200	2.050	0.8318
M6	SMA type 2	2	-0.920	1.2869	-0.920	-1.830	-0.010	1.820	-1.830	-0.010	0.5000
M6	SMA type 3	14	-0.094	1.6325	-0.255	-0.860	1.660	2.520	-3.960	1.940	0.7905
M6	Total	16	-0.198	1.5810	-0.255	-0.940	1.045	1.985	-3.960	1.940	0.4545
M7	SMA type 3	8	0.294	1.0520	0.075	-0.430	0.795	1.225	-0.880	2.350	1.0000
M7	Total	8	0.294	1.0520	0.075	-0.430	0.795	1.225	-0.880	2.350	1.0000
M8	SMA type 3	2	0.190	2.8284	0.190	-1.810	2.190	4.000	-1.810	2.190	1.0000
M8	Total	2	0.190	2.8284	0.190	-1.810	2.190	4.000	-1.810	2.190	1.0000
Last available	SMA type 2	13	0.061	1.2204	0.180	-0.180	0.590	0.770	-2.350	2.440	0.5811
Last available	SMA type 3	35	-0.160	1.4666	-0.090	-0.880	0.450	1.330	-3.970	2.930	0.7359
Last available	Total	48	-0.100	1.3950	0.000	-0.710	0.490	1.200	-3.970	2.930	1.0000

***p-value of sign test**

Table S2f. Change from baseline (L1): Platelet count [number/nL].

Injection	SMA type	N	Mean	Std Dev	Median	Lower Quartile	Upper Quartile	Quartile Range	Min	Max	p-value*
L2	SMA type 2	11	-7.000	49.9480	-12.000	-46.000	1.000	47.000	-57.000	117.000	0.5488
L2	SMA type 3	21	-2.333	35.6810	-4.000	-22.000	15.000	37.000	-68.000	74.000	1.0000
L2	Total	32	-3.938	40.3884	-8.000	-35.000	13.500	48.500	-68.000	117.000	0.5966
L3	SMA type 2	11	-22.909	67.3253	-20.000	-71.000	3.000	74.000	-109.000	137.000	0.2266
L3	SMA type 3	21	-2.381	35.0777	-4.000	-23.000	16.000	39.000	-71.000	83.000	0.6636
L3	Total	32	-9.438	48.5193	-9.500	-35.000	14.500	49.500	-109.000	137.000	0.2153
L4	SMA type 2	11	-8.182	109.3836	7.000	-73.000	44.000	117.000	-265.000	148.000	0.5488
L4	SMA type 3	20	3.350	31.3944	-4.500	-18.000	20.500	38.500	-37.000	91.000	0.8238
L4	Total	31	-0.742	68.1464	3.000	-19.000	25.000	44.000	-265.000	148.000	1.0000
M1	SMA type 2	10	8.500	66.4584	0.500	-23.000	32.000	55.000	-114.000	123.000	1.0000
M1	SMA type 3	27	11.407	42.8308	4.000	-18.000	29.000	47.000	-53.000	139.000	0.8450
M1	Total	37	10.622	49.3031	4.000	-20.000	29.000	49.000	-114.000	139.000	0.8679
M2	SMA type 2	11	-16.273	65.0278	-25.000	-49.000	20.000	69.000	-135.000	125.000	0.5488
M2	SMA type 3	26	8.269	54.5253	0.000	-14.000	19.000	33.000	-78.000	172.000	1.0000
M2	Total	37	0.973	58.0390	-2.000	-25.000	19.000	44.000	-135.000	172.000	0.7359
M3	SMA type 2	10	-10.500	95.4908	-19.000	-68.000	12.000	80.000	-159.000	212.000	0.7539
M3	SMA type 3	18	-4.944	44.5110	2.500	-26.000	20.000	46.000	-119.000	95.000	0.8145
M3	Total	28	-6.929	65.5308	-1.000	-34.500	19.500	54.000	-159.000	212.000	1.0000
M4	SMA type 2	8	-38.750	98.3198	-32.000	-101.500	13.000	114.500	-195.000	126.000	1.0000
M4	SMA type 3	15	8.000	36.4319	4.000	-24.000	20.000	44.000	-41.000	80.000	0.6072
M4	Total	23	-8.261	66.6239	4.000	-40.000	19.000	59.000	-195.000	126.000	0.6776
M5	SMA type 2	6	-60.333	60.2849	-56.000	-117.000	-7.000	110.000	-139.000	13.000	0.2188
M5	SMA type 3	16	-1.188	37.8104	2.500	-25.500	26.000	51.500	-73.000	62.000	0.8036
M5	Total	22	-17.318	51.1211	-8.000	-40.000	13.000	53.000	-139.000	62.000	0.8318
M6	SMA type 2	2	-83.000	4.2426	-83.000	-86.000	-80.000	6.000	-86.000	-80.000	0.5000
M6	SMA type 3	14	-7.214	44.4543	12.000	-48.000	27.000	75.000	-94.000	54.000	0.7905
M6	Total	16	-16.688	48.8258	-4.000	-54.500	25.500	80.000	-94.000	54.000	1.0000
M7	SMA type 3	8	0.375	16.8008	-2.000	-13.500	16.500	30.000	-21.000	22.000	1.0000
M7	Total	8	0.375	16.8008	-2.000	-13.500	16.500	30.000	-21.000	22.000	1.0000
M8	SMA type 3	2	16.000	9.8995	16.000	9.000	23.000	14.000	9.000	23.000	0.5000
M8	Total	2	16.000	9.8995	16.000	9.000	23.000	14.000	9.000	23.000	0.5000
Last available	SMA type 2	13	-39.000	95.3904	-33.000	-101.000	1.000	102.000	-159.000	212.000	0.2668
Last available	SMA type 3	35	2.714	37.3076	3.000	-17.000	23.000	40.000	-94.000	95.000	0.8642
Last available	Total	48	-8.583	60.6717	-4.500	-28.000	22.000	50.000	-159.000	212.000	0.7709

***p-value of sign test**

Table S2g. Change from baseline (L1): International normalized ratio (INR).

Injection	SMA type	N	Mean	Std Dev	Median	Lower Quartile	Upper Quartile	Quartile Range	Min	Max	p-value*
L2	SMA type 2	11	0.008	0.0565	0.020	-0.050	0.050	0.100	-0.070	0.100	1.0000
L2	SMA type 3	12	0.004	0.0323	0.015	-0.025	0.025	0.050	-0.050	0.050	0.5488
L2	Total	23	0.006	0.0445	0.020	-0.030	0.040	0.070	-0.070	0.100	0.5235
L3	SMA type 2	9	0.002	0.0524	0.020	-0.040	0.030	0.070	-0.060	0.090	1.0000
L3	SMA type 3	11	-0.006	0.0406	-0.010	-0.030	0.000	0.030	-0.050	0.100	0.1797
L3	Total	20	-0.003	0.0452	-0.010	-0.035	0.020	0.055	-0.060	0.100	0.4807
L4	SMA type 2	10	0.011	0.0489	0.010	-0.020	0.040	0.060	-0.060	0.110	1.0000
L4	SMA type 3	11	-0.002	0.0334	0.000	-0.030	0.010	0.040	-0.040	0.080	1.0000
L4	Total	21	0.004	0.0409	0.000	-0.020	0.020	0.040	-0.060	0.110	1.0000
M1	SMA type 2	10	0.022	0.0607	0.000	-0.020	0.070	0.090	-0.070	0.120	1.0000
M1	SMA type 3	10	0.003	0.0320	0.005	-0.030	0.030	0.060	-0.040	0.050	1.0000
M1	Total	20	0.013	0.0482	0.000	-0.020	0.035	0.055	-0.070	0.120	0.8036
M2	SMA type 2	11	0.014	0.0590	0.000	-0.030	0.080	0.110	-0.070	0.090	1.0000
M2	SMA type 3	8	0.028	0.0324	0.020	0.020	0.045	0.025	-0.030	0.080	0.0703
M2	Total	19	0.019	0.0489	0.020	-0.020	0.070	0.090	-0.070	0.090	0.2379
M3	SMA type 2	9	0.010	0.0574	0.000	-0.020	0.070	0.090	-0.070	0.090	1.0000
M3	SMA type 3	3	0.017	0.0513	0.030	-0.040	0.060	0.100	-0.040	0.060	1.0000
M3	Total	12	0.012	0.0537	0.010	-0.030	0.065	0.095	-0.070	0.090	1.0000
M4	SMA type 2	8	0.008	0.0385	0.005	-0.020	0.030	0.050	-0.040	0.070	0.6875
M4	SMA type 3	3	0.017	0.0153	0.020	0.000	0.030	0.030	0.000	0.030	0.5000
M4	Total	11	0.010	0.0332	0.010	0.000	0.030	0.030	-0.040	0.070	0.2891
M5	SMA type 2	6	0.002	0.0319	-0.005	-0.030	0.040	0.070	-0.030	0.040	1.0000
M5	SMA type 3	3	0.017	0.0379	0.000	-0.010	0.060	0.070	-0.010	0.060	1.0000
M5	Total	9	0.007	0.0324	0.000	-0.010	0.040	0.050	-0.030	0.060	1.0000
M6	SMA type 2	2	0.010	0.0141	0.010	0.000	0.020	0.020	0.000	0.020	1.0000
M6	SMA type 3	3	0.057	0.0611	0.070	-0.010	0.110	0.120	-0.010	0.110	1.0000
M6	Total	5	0.038	0.0507	0.020	0.000	0.070	0.070	-0.010	0.110	0.6250
M7	SMA type 3	1	0.050	.	0.050	0.050	0.050	0.000	0.050	0.050	1.0000
M7	Total	1	0.050	.	0.050	0.050	0.050	0.000	0.050	0.050	1.0000
Last available	SMA type 2	12	0.014	0.0326	0.005	-0.010	0.040	0.050	-0.030	0.080	0.7539
Last available	SMA type 3	13	0.012	0.0426	0.000	-0.020	0.030	0.050	-0.030	0.110	1.0000
Last available	Total	25	0.013	0.0374	0.000	-0.020	0.040	0.060	-0.030	0.110	0.8318
***p-value of sign test**											

Table S2h. Change from baseline (L1): Activated partial thromboplastin time (aPTT) [s].

Injection	SMA type	N	Mean	Std Dev	Median	Lower Quartile	Upper Quartile	Quartile Range	Min	Max	p-value*
L2	SMA type 2	11	0.000	1.1636	-0.100	-1.000	1.100	2.100	-1.700	1.800	1.0000
L2	SMA type 3	11	-0.373	0.7643	-0.400	-1.000	0.100	1.100	-1.200	1.300	0.3438
L2	Total	22	-0.186	0.9795	-0.200	-1.000	0.300	1.300	-1.700	1.800	0.3833
L3	SMA type 2	9	-0.844	2.1448	-1.000	-1.300	0.200	1.500	-5.600	1.800	0.5078
L3	SMA type 3	11	-0.991	2.2133	-0.100	-2.100	0.400	2.500	-5.900	1.900	0.7539
L3	Total	20	-0.925	2.1262	-0.700	-1.550	0.350	1.900	-5.900	1.900	0.3593
L4	SMA type 2	10	-0.210	1.7214	0.250	-0.600	0.500	1.100	-4.700	1.800	0.3438
L4	SMA type 3	11	-0.791	0.8983	-0.900	-1.300	-0.300	1.000	-2.500	1.100	0.0117
L4	Total	21	-0.514	1.3510	-0.600	-1.000	0.400	1.400	-4.700	1.800	0.3833
M1	SMA type 2	10	-0.280	2.0330	-0.550	-1.500	1.600	3.100	-4.200	2.400	1.0000
M1	SMA type 3	10	-1.150	1.8374	-1.350	-2.200	-0.700	1.500	-4.300	2.100	0.1094
M1	Total	20	-0.715	1.9381	-1.200	-1.650	1.250	2.900	-4.300	2.400	0.2632
M2	SMA type 2	11	0.418	1.9626	0.600	-1.600	2.400	4.000	-2.400	2.500	1.0000
M2	SMA type 3	8	0.425	1.6926	0.850	-0.100	1.450	1.550	-3.200	2.200	0.2891
M2	Total	19	0.421	1.8039	0.800	-1.000	2.100	3.100	-3.200	2.500	0.3593
M3	SMA type 2	9	-1.067	2.6608	-0.200	-2.900	1.200	4.100	-5.500	2.000	1.0000
M3	SMA type 3	3	0.400	1.7776	-0.200	-1.000	2.400	3.400	-1.000	2.400	1.0000
M3	Total	12	-0.700	2.4827	-0.200	-2.450	1.200	3.650	-5.500	2.400	0.7744
M4	SMA type 2	8	-1.375	1.8375	-1.950	-2.900	0.200	3.100	-3.300	1.600	0.2891
M4	SMA type 3	3	-0.733	0.4163	-0.600	-1.200	-0.400	0.800	-1.200	-0.400	0.2500
M4	Total	11	-1.200	1.5773	-1.200	-2.800	-0.100	2.700	-3.300	1.600	0.0654
M5	SMA type 2	6	-0.533	1.0912	-0.450	-1.600	0.500	2.100	-1.800	0.600	1.0000
M5	SMA type 3	3	-1.767	0.9866	-1.300	-2.900	-1.100	1.800	-2.900	-1.100	0.2500
M5	Total	9	-0.944	1.1695	-1.100	-1.600	0.200	1.800	-2.900	0.600	0.5078
M6	SMA type 2	2	-3.250	3.1820	-3.250	-5.500	-1.000	4.500	-5.500	-1.000	0.5000
M6	SMA type 3	3	-0.600	1.0149	-0.400	-1.700	0.300	2.000	-1.700	0.300	1.0000
M6	Total	5	-1.660	2.2700	-1.000	-1.700	-0.400	1.300	-5.500	0.300	0.3750
M7	SMA type 3	1	-0.700	.	-0.700	-0.700	-0.700	0.000	-0.700	-0.700	1.0000
M7	Total	1	-0.700	.	-0.700	-0.700	-0.700	0.000	-0.700	-0.700	1.0000
Last available	SMA type 2	12	-2.117	1.9423	-1.600	-3.150	-1.000	2.150	-5.500	0.500	0.0386
Last available	SMA type 3	13	-0.700	1.6119	-0.700	-1.700	-0.100	1.600	-3.200	2.200	0.0923
Last available	Total	25	-1.380	1.8841	-1.000	-2.500	-0.300	2.200	-5.500	2.200	0.0041

***p-value of sign test**

Table S2i. Change from baseline (L1): Creatinine [mg/dL].

Injection	SMA type	N	Mean	Std Dev	Median	Lower Quartile	Upper Quartile	Quartile Range	Min	Max	p-value*
L2	SMA type 2	10	-0.025	0.0448	-0.025	-0.070	0.010	0.080	-0.090	0.050	0.5078
L2	SMA type 3	21	0.043	0.1225	0.030	0.000	0.050	0.050	-0.170	0.390	0.0963
L2	Total	31	0.021	0.1080	0.010	-0.030	0.040	0.070	-0.170	0.390	0.4421
L3	SMA type 2	11	-0.011	0.0614	-0.020	-0.040	0.020	0.060	-0.130	0.120	0.5488
L3	SMA type 3	19	-0.007	0.0909	0.010	-0.050	0.040	0.090	-0.210	0.160	0.8145
L3	Total	30	-0.009	0.0802	-0.005	-0.040	0.030	0.070	-0.210	0.160	1.0000
L4	SMA type 2	10	-0.004	0.0695	0.010	-0.050	0.030	0.080	-0.120	0.110	0.7539
L4	SMA type 3	19	-0.017	0.0682	0.000	-0.060	0.040	0.100	-0.150	0.070	1.0000
L4	Total	29	-0.012	0.0677	0.010	-0.050	0.040	0.090	-0.150	0.110	0.8506
M1	SMA type 2	10	-0.001	0.0584	0.015	-0.030	0.030	0.060	-0.110	0.090	0.7539
M1	SMA type 3	25	-0.033	0.0949	-0.010	-0.080	0.030	0.110	-0.300	0.110	0.8388
M1	Total	35	-0.024	0.0865	0.000	-0.070	0.030	0.100	-0.300	0.110	1.0000
M2	SMA type 2	10	0.013	0.1237	-0.005	-0.070	0.040	0.110	-0.110	0.330	1.0000
M2	SMA type 3	25	-0.032	0.0745	-0.020	-0.060	0.010	0.070	-0.220	0.080	0.0433
M2	Total	35	-0.019	0.0916	-0.020	-0.060	0.020	0.080	-0.220	0.330	0.0576
M3	SMA type 2	9	-0.018	0.0959	0.010	-0.060	0.020	0.080	-0.170	0.160	1.0000
M3	SMA type 3	17	-0.032	0.0706	-0.010	-0.080	0.020	0.100	-0.190	0.070	0.4240
M3	Total	26	-0.027	0.0786	-0.005	-0.080	0.020	0.100	-0.190	0.160	0.6776
M4	SMA type 2	8	-0.019	0.1214	-0.005	-0.110	0.090	0.200	-0.220	0.120	1.0000
M4	SMA type 3	15	-0.015	0.0704	-0.010	-0.050	0.040	0.090	-0.170	0.120	0.4240
M4	Total	23	-0.017	0.0886	-0.010	-0.060	0.050	0.110	-0.220	0.120	0.5235
M5	SMA type 2	6	0.048	0.1689	0.045	-0.080	0.210	0.290	-0.150	0.220	1.0000
M5	SMA type 3	16	-0.023	0.0632	-0.010	-0.065	0.020	0.085	-0.140	0.070	0.4545
M5	Total	22	-0.003	0.1034	-0.010	-0.080	0.050	0.130	-0.150	0.220	0.5235
M6	SMA type 2	2	-0.125	0.0495	-0.125	-0.160	-0.090	0.070	-0.160	-0.090	0.5000
M6	SMA type 3	14	-0.084	0.1113	-0.100	-0.160	-0.030	0.130	-0.270	0.170	0.0574
M6	Total	16	-0.089	0.1054	-0.100	-0.160	-0.040	0.120	-0.270	0.170	0.0213
M7	SMA type 3	8	-0.095	0.0661	-0.115	-0.135	-0.055	0.080	-0.170	0.020	0.0703
M7	Total	8	-0.095	0.0661	-0.115	-0.135	-0.055	0.080	-0.170	0.020	0.0703
M8	SMA type 3	2	-0.130	0.1556	-0.130	-0.240	-0.020	0.220	-0.240	-0.020	0.5000
M8	Total	2	-0.130	0.1556	-0.130	-0.240	-0.020	0.220	-0.240	-0.020	0.5000
Last available	SMA type 2	13	-0.041	0.1159	-0.080	-0.110	0.000	0.110	-0.170	0.210	0.1460
Last available	SMA type 3	33	-0.109	0.0885	-0.120	-0.160	-0.050	0.110	-0.300	0.070	<.0001
Last available	Total	46	-0.090	0.1006	-0.100	-0.150	-0.040	0.110	-0.300	0.210	<.0001

***p-value of sign test**

Table S2j. Change from baseline (L1): Urea nitrogen [mg/dL].

Injection	SMA type	N	Mean	Std Dev	Median	Lower Quartile	Upper Quartile	Quartile Range	Min	Max	p-value*
L2	SMA type 2	10	0.400	3.5963	0.000	-1.000	1.000	2.000	-5.000	9.000	1.0000
L2	SMA type 3	21	0.881	3.1619	1.000	-1.000	3.000	4.000	-4.000	9.000	0.3593
L2	Total	31	0.726	3.2554	0.500	-1.000	2.000	3.000	-5.000	9.000	0.4421
L3	SMA type 2	11	0.182	2.7863	0.000	0.000	1.000	1.000	-7.000	5.000	0.2188
L3	SMA type 3	19	0.289	2.6942	0.000	-2.000	2.000	4.000	-4.000	7.000	1.0000
L3	Total	30	0.250	2.6805	0.000	-1.000	1.000	2.000	-7.000	7.000	0.5413
L4	SMA type 2	10	1.400	2.5033	1.000	1.000	1.000	0.000	-1.000	8.000	0.1094
L4	SMA type 3	19	-0.895	3.1667	-2.000	-3.000	2.000	5.000	-6.400	5.000	0.2379
L4	Total	29	-0.103	3.1134	0.000	-2.000	1.000	3.000	-6.400	8.000	1.0000
M1	SMA type 2	10	1.570	2.8449	2.000	-1.000	3.000	4.000	-3.000	7.000	0.3438
M1	SMA type 3	24	-0.754	2.0862	0.000	-3.000	0.450	3.450	-4.200	2.400	0.3323
M1	Total	34	-0.071	2.5290	0.000	-1.000	2.000	3.000	-4.200	7.000	1.0000
M2	SMA type 2	10	2.670	4.6452	1.000	0.000	5.000	5.000	-2.000	14.000	0.1797
M2	SMA type 3	25	-0.280	3.7958	0.000	-2.000	2.000	4.000	-11.000	5.000	0.8318
M2	Total	35	0.563	4.2084	1.000	-2.000	2.000	4.000	-11.000	14.000	0.2810
M3	SMA type 2	9	1.856	2.9728	2.000	0.000	3.000	3.000	-2.000	7.000	0.4531
M3	SMA type 3	17	0.200	3.1979	1.000	-1.700	2.000	3.700	-9.000	4.000	0.4240
M3	Total	26	0.773	3.1651	1.000	-1.300	3.000	4.300	-9.000	7.000	0.1892
M4	SMA type 2	8	1.450	4.4782	1.000	0.200	4.100	3.900	-7.000	8.000	0.2891
M4	SMA type 3	15	-0.600	3.1803	-2.000	-3.000	2.000	5.000	-5.000	4.000	1.0000
M4	Total	23	0.113	3.7167	1.000	-2.000	2.200	4.200	-7.000	8.000	0.6776
M5	SMA type 2	6	2.583	1.9600	3.000	2.000	4.000	2.000	-1.000	4.500	0.2188
M5	SMA type 3	16	-0.225	3.4847	0.500	-3.000	2.500	5.500	-8.000	4.000	1.0000
M5	Total	22	0.541	3.3507	1.200	-1.000	3.000	4.000	-8.000	4.500	0.3833
M6	SMA type 2	2	2.050	1.2021	2.050	1.200	2.900	1.700	1.200	2.900	0.5000
M6	SMA type 3	14	-0.657	2.9586	-1.500	-3.000	2.000	5.000	-4.000	5.000	0.1796
M6	Total	16	-0.319	2.9219	-0.850	-3.000	2.450	5.450	-4.000	5.000	0.4545
M7	SMA type 3	8	0.013	3.4967	-0.650	-2.800	2.850	5.650	-4.300	5.600	1.0000
M7	Total	8	0.013	3.4967	-0.650	-2.800	2.850	5.650	-4.300	5.600	1.0000
M8	SMA type 3	2	-6.900	7.6368	-6.900	-12.300	-1.500	10.800	-12.300	-1.500	0.5000
M8	Total	2	-6.900	7.6368	-6.900	-12.300	-1.500	10.800	-12.300	-1.500	0.5000
Last available	SMA type 2	13	1.469	2.3991	1.000	-0.600	3.000	3.600	-2.000	5.700	0.2668
Last available	SMA type 3	33	-0.524	3.7102	-0.600	-2.600	2.100	4.700	-12.300	5.600	0.5847
Last available	Total	46	0.039	3.4853	0.000	-1.700	2.900	4.600	-12.300	5.700	1.0000

***p-value of sign test**

Table S2k. Change from baseline (L1): Aspartate aminotransferase (AST) [U/L].

Injection	SMA type	N	Mean	Std Dev	Median	Lower Quartile	Upper Quartile	Quartile Range	Min	Max	p-value*
L2	SMA type 2	10	-5.100	6.8710	-2.500	-9.000	0.000	9.000	-20.000	2.000	0.1797
L2	SMA type 3	21	-3.190	5.4002	-3.000	-7.000	-1.000	6.000	-14.000	8.000	0.0266
L2	Total	31	-3.806	5.8675	-3.000	-8.000	0.000	8.000	-20.000	8.000	0.0052
L3	SMA type 2	11	-2.818	7.2913	-3.000	-7.000	3.000	10.000	-15.000	9.000	0.5488
L3	SMA type 3	19	-2.000	6.8069	-3.000	-6.000	3.000	9.000	-16.000	13.000	0.3593
L3	Total	30	-2.300	6.8740	-3.000	-6.000	3.000	9.000	-16.000	13.000	0.2005
L4	SMA type 2	10	-1.300	6.0562	-1.500	-6.000	3.000	9.000	-10.000	9.000	0.5078
L4	SMA type 3	19	-1.000	7.1414	-1.000	-4.000	5.000	9.000	-17.000	13.000	0.3593
L4	Total	29	-1.103	6.6780	-1.000	-4.000	3.000	7.000	-17.000	13.000	0.1849
M1	SMA type 2	10	-5.200	7.0364	-3.000	-5.000	-3.000	2.000	-21.000	3.000	0.0391
M1	SMA type 3	24	-2.167	7.4930	-0.500	-5.500	3.500	9.000	-30.000	6.000	0.6636
M1	Total	34	-3.059	7.3894	-3.000	-5.000	3.000	8.000	-30.000	6.000	0.0987
M2	SMA type 2	9	-2.000	5.6569	-3.000	-4.000	0.000	4.000	-12.000	8.000	0.2891
M2	SMA type 3	25	0.880	16.9172	-1.000	-3.000	4.000	7.000	-30.000	70.000	0.8388
M2	Total	34	0.118	14.7500	-1.500	-3.000	4.000	7.000	-30.000	70.000	0.3771
M3	SMA type 2	9	-4.333	4.4159	-6.000	-7.000	-1.000	6.000	-11.000	2.000	0.1797
M3	SMA type 3	17	1.176	16.4440	1.000	-7.000	3.000	10.000	-23.000	52.000	1.0000
M3	Total	26	-0.731	13.6545	-1.000	-7.000	3.000	10.000	-23.000	52.000	0.5572
M4	SMA type 2	8	-5.625	7.5392	-3.000	-8.000	-2.000	6.000	-22.000	3.000	0.0703
M4	SMA type 3	15	0.667	10.1957	0.000	-4.000	6.000	10.000	-20.000	20.000	1.0000
M4	Total	23	-1.522	9.6760	-2.000	-4.000	5.000	9.000	-22.000	20.000	0.3833
M5	SMA type 2	6	-4.000	10.3537	-3.000	-9.000	3.000	12.000	-21.000	9.000	0.6875
M5	SMA type 3	16	4.188	15.0431	1.500	-5.000	10.000	15.000	-21.000	39.000	0.6072
M5	Total	22	1.955	14.1807	0.500	-5.000	6.000	11.000	-21.000	39.000	1.0000
M6	SMA type 2	2	-3.000	2.8284	-3.000	-5.000	-1.000	4.000	-5.000	-1.000	0.5000
M6	SMA type 3	14	1.500	10.2188	3.000	-4.000	7.000	11.000	-20.000	17.000	0.4240
M6	Total	16	0.938	9.6642	2.000	-4.500	6.500	11.000	-20.000	17.000	0.8036
M7	SMA type 3	8	1.875	13.3463	2.500	-4.000	10.500	14.500	-23.000	20.000	1.0000
M7	Total	8	1.875	13.3463	2.500	-4.000	10.500	14.500	-23.000	20.000	1.0000
M8	SMA type 3	2	-13.500	12.0208	-13.500	-22.000	-5.000	17.000	-22.000	-5.000	0.5000
M8	Total	2	-13.500	12.0208	-13.500	-22.000	-5.000	17.000	-22.000	-5.000	0.5000
Last available	SMA type 2	13	-1.615	5.4089	-3.000	-5.000	-1.000	4.000	-9.000	9.000	0.0923
Last available	SMA type 3	33	1.182	9.3690	1.000	-4.000	5.000	9.000	-22.000	27.000	1.0000
Last available	Total	46	0.391	8.4761	-1.000	-5.000	5.000	10.000	-22.000	27.000	0.4614

***p-value of sign test**

Table S2l. Change from baseline (L1): Alanine aminotransferase (ALT) [U/L].

Injection	SMA type	N	Mean	Std Dev	Median	Lower Quartile	Upper Quartile	Quartile Range	Min	Max	p-value*
L2	SMA type 2	10	-4.600	13.0231	0.500	-12.000	3.000	15.000	-31.000	13.000	1.0000
L2	SMA type 3	21	-4.429	6.4929	-4.000	-7.000	-2.000	5.000	-22.000	10.000	0.0015
L2	Total	31	-4.484	8.8877	-4.000	-8.000	1.000	9.000	-31.000	13.000	0.0161
L3	SMA type 2	11	-5.727	9.9909	-2.000	-17.000	2.000	19.000	-24.000	4.000	0.5078
L3	SMA type 3	19	1.263	14.3482	0.000	-11.000	11.000	22.000	-20.000	34.000	1.0000
L3	Total	30	-1.300	13.1887	-1.000	-11.000	2.000	13.000	-24.000	34.000	0.7011
L4	SMA type 2	10	-3.900	13.5929	-4.500	-17.000	6.000	23.000	-21.000	17.000	0.7539
L4	SMA type 3	19	-5.474	14.5921	-2.000	-11.000	1.000	12.000	-50.000	21.000	0.2379
L4	Total	29	-4.931	14.0304	-2.000	-11.000	2.000	13.000	-50.000	21.000	0.1849
M1	SMA type 2	10	-7.800	11.0534	-4.000	-8.000	0.000	8.000	-32.000	2.000	0.0703
M1	SMA type 3	24	-4.792	12.3323	-4.500	-12.000	4.000	16.000	-35.000	16.000	0.2100
M1	Total	34	-5.676	11.8851	-4.500	-11.000	2.000	13.000	-35.000	16.000	0.0294
M2	SMA type 2	9	-3.889	9.4001	-1.000	-11.000	2.000	13.000	-21.000	9.000	1.0000
M2	SMA type 3	25	1.840	34.5467	-2.000	-11.000	9.000	20.000	-37.000	147.000	0.8388
M2	Total	34	0.324	29.9330	-1.500	-11.000	7.000	18.000	-37.000	147.000	0.7283
M3	SMA type 2	9	-4.667	8.6747	-3.000	-9.000	1.000	10.000	-23.000	5.000	0.5078
M3	SMA type 3	17	2.529	32.7912	3.000	-6.000	9.000	15.000	-54.000	110.000	1.0000
M3	Total	26	0.038	26.9154	-2.000	-9.000	5.000	14.000	-54.000	110.000	0.8450
M4	SMA type 2	8	-11.375	22.9031	-2.500	-14.000	0.000	14.000	-64.000	6.000	0.2891
M4	SMA type 3	15	4.733	22.1439	0.000	-4.000	16.000	20.000	-33.000	44.000	1.0000
M4	Total	23	-0.870	23.2483	-2.000	-5.000	15.000	20.000	-64.000	44.000	0.5235
M5	SMA type 2	6	-8.167	34.9767	1.000	-6.000	5.000	11.000	-76.000	26.000	1.0000
M5	SMA type 3	16	6.625	25.4293	5.000	-5.000	19.500	24.500	-40.000	75.000	0.8036
M5	Total	22	2.591	28.2601	2.000	-6.000	16.000	22.000	-76.000	75.000	0.6636
M6	SMA type 2	2	-41.500	45.9619	-41.500	-74.000	-9.000	65.000	-74.000	-9.000	0.5000
M6	SMA type 3	14	1.714	17.0809	4.000	-12.000	14.000	26.000	-31.000	33.000	0.7905
M6	Total	16	-3.688	24.7298	0.000	-12.500	11.000	23.500	-74.000	33.000	1.0000
M7	SMA type 3	8	2.125	30.7638	4.000	-16.500	13.500	30.000	-45.000	60.000	1.0000
M7	Total	8	2.125	30.7638	4.000	-16.500	13.500	30.000	-45.000	60.000	1.0000
M8	SMA type 3	2	-24.000	31.1127	-24.000	-46.000	-2.000	44.000	-46.000	-2.000	0.5000
M8	Total	2	-24.000	31.1127	-24.000	-46.000	-2.000	44.000	-46.000	-2.000	0.5000
Last available	SMA type 2	13	-4.692	23.6057	0.000	-6.000	5.000	11.000	-74.000	26.000	1.0000
Last available	SMA type 3	33	-1.636	18.8046	-2.000	-12.000	8.000	20.000	-46.000	60.000	0.2153
Last available	Total	46	-2.500	20.0497	-2.000	-12.000	6.000	18.000	-74.000	60.000	0.3604

***p-value of sign test**

Table S2m. Change from baseline (L1): Gamma-glutamyltransferase (GGT) [U/L].

Injection	SMA type	N	Mean	Std Dev	Median	Lower Quartile	Upper Quartile	Quartile Range	Min	Max	p-value*
L2	SMA type 2	10	-3.300	11.5859	-2.000	-4.000	4.000	8.000	-34.000	7.000	0.7539
L2	SMA type 3	21	-7.714	15.9848	-2.000	-7.000	0.000	7.000	-61.000	5.000	0.0309
L2	Total	31	-6.290	14.6633	-2.000	-7.000	1.000	8.000	-61.000	7.000	0.0357
L3	SMA type 2	11	0.636	5.0452	3.000	-1.000	4.000	5.000	-12.000	5.000	0.5488
L3	SMA type 3	19	0.211	20.6306	3.000	-3.000	9.000	12.000	-76.000	26.000	0.2379
L3	Total	30	0.367	16.5227	3.000	-3.000	4.000	7.000	-76.000	26.000	0.1360
L4	SMA type 2	10	4.800	10.2610	3.000	-4.000	6.000	10.000	-6.000	24.000	0.7539
L4	SMA type 3	19	-10.105	27.9799	-1.000	-9.000	4.000	13.000	-97.000	11.000	0.6291
L4	Total	29	-4.966	24.2715	0.000	-4.000	4.000	8.000	-97.000	24.000	1.0000
M1	SMA type 2	10	-4.400	16.6012	0.000	-4.000	5.000	9.000	-50.000	6.000	1.0000
M1	SMA type 3	24	-8.667	19.7499	-3.500	-8.500	0.000	8.500	-93.000	7.000	0.0169
M1	Total	34	-7.412	18.7328	-3.000	-7.000	2.000	9.000	-93.000	7.000	0.0428
M2	SMA type 2	9	5.000	7.0356	4.000	1.000	9.000	8.000	-4.000	20.000	0.1797
M2	SMA type 3	25	-4.520	22.9821	1.000	-6.000	4.000	10.000	-83.000	27.000	1.0000
M2	Total	34	-2.000	20.3544	1.500	-4.000	5.000	9.000	-83.000	27.000	0.3915
M3	SMA type 2	9	6.667	16.0390	0.000	-2.000	4.000	6.000	-7.000	38.000	1.0000
M3	SMA type 3	17	-9.412	26.1774	-1.000	-9.000	3.000	12.000	-83.000	21.000	0.8036
M3	Total	26	-3.846	24.1192	-0.500	-7.000	4.000	11.000	-83.000	38.000	0.8388
M4	SMA type 2	8	4.375	19.5444	-2.000	-5.500	6.500	12.000	-13.000	50.000	0.7266
M4	SMA type 3	15	0.667	28.7270	-1.000	-11.000	10.000	21.000	-50.000	61.000	1.0000
M4	Total	23	1.957	25.4942	-2.000	-8.000	7.000	15.000	-50.000	61.000	0.6776
M5	SMA type 2	6	4.667	35.9036	-1.500	-7.000	5.000	12.000	-38.000	71.000	1.0000
M5	SMA type 3	16	1.375	37.0889	1.000	-12.000	18.000	30.000	-86.000	89.000	1.0000
M5	Total	22	2.273	35.9407	0.000	-7.000	18.000	25.000	-86.000	89.000	1.0000
M6	SMA type 2	2	-20.000	22.6274	-20.000	-36.000	-4.000	32.000	-36.000	-4.000	0.5000
M6	SMA type 3	14	4.643	27.0060	2.500	-4.000	5.000	9.000	-46.000	77.000	0.4240
M6	Total	16	1.563	27.1489	1.500	-7.500	4.500	12.000	-46.000	77.000	0.8036
M7	SMA type 3	8	5.000	30.4209	0.000	-8.500	21.500	30.000	-43.000	57.000	1.0000
M7	Total	8	5.000	30.4209	0.000	-8.500	21.500	30.000	-43.000	57.000	1.0000
M8	SMA type 3	2	1.500	21.9203	1.500	-14.000	17.000	31.000	-14.000	17.000	1.0000
M8	Total	2	1.500	21.9203	1.500	-14.000	17.000	31.000	-14.000	17.000	1.0000
Last available	SMA type 2	13	3.538	23.7122	-2.000	-4.000	5.000	9.000	-36.000	71.000	1.0000
Last available	SMA type 3	33	-6.242	22.2008	-1.000	-11.000	3.000	14.000	-86.000	37.000	0.4869
Last available	Total	46	-3.478	22.8091	-1.500	-7.000	4.000	11.000	-86.000	71.000	0.4614
***p-value of sign test**											
